# Rare variant contribution to cholestatic liver disease in a South Asian population in the United Kingdom

**DOI:** 10.1038/s41598-023-33391-w

**Published:** 2023-05-19

**Authors:** Julia Zöllner, Sarah Finer, Kenneth J. Linton, Genes and Health Research Team, Shaheen Akhtar, Mohammad Anwar, Elena Arciero, Samina Ashraf, Saeed Bidi, Gerome Breen, James Broster, Raymond Chung, David Collier, Charles J. Curtis, Shabana Chaudhary, Megan Clinch, Grainne Colligan, Panos Deloukas, Ceri Durham, Faiza Durrani, Fabiola Eto, Sarah Finer, Joseph Gafton, Ana Angel Garcia, Chris Griffiths, Joanne Harvey, Teng Heng, Sam Hodgson, Qin Qin Huang, Matt Hurles, Karen A. Hunt, Shapna Hussain, Kamrul Islam, Vivek Iyer, Ben Jacobs, Ahsan Khan, Cath Lavery, Sang Hyuck Lee, Robin Lerner, Daniel MacArthur, Daniel Malawsky, Hilary Martin, Dan Mason, Rohini Mathur, Mohammed Bodrul Mazid, John McDermott, Caroline Morton, Bill Newman, Elizabeth Owor, Asma Qureshi, Samiha Rahman, Shwetha Ramachandrappa, Mehru Reza, Jessry Russell, Nishat Safa, Miriam Samuel, Michael Simpson, John Solly, Marie Spreckley, Daniel Stow, Michael Taylor, Richard C. Trembath, Karen Tricker, Nasir Uddin, David A. van Heel, Klaudia Walter, Caroline Winckley, Suzanne Wood, John Wright, Julia Zöllner, David A. van Heel, Catherine Williamson, Peter H. Dixon

**Affiliations:** 1 University College London, London, UK; 2 Institute for Population Health Sciences, Barts and the London School of Medicine and Dentistry, Queen Mary University of London, London, UK; 3 Centre for Cell Biology and Cutaneous Research, Blizard Institute, Barts and the London School of Medicine and Dentistry, Queen Mary University of London, London, UK; 4 Blizard Institute, Barts and the London School of Medicine and Dentistry, Queen Mary University of London, London, UK; 5 Department of Women and Children’s Health, School of Life Course Sciences, FOLSM, King’s College London, 2.30W Hodgkin Building, Guy’s Campus, London, SE1 1UL UK; 6 Wellcome Sanger Institute, London, UK; 7 Social Action for Health, London, UK; 8 Bradford Teaching Hospitals, Bradford, UK; 9 Queen Mary University of London, London, UK; 10 King’s College London, London, UK; 11 Garvan Institute of Medical Research, Darlinghurst, Australia; 12 Born in Bradford, Bradford, UK; 13 University of Manchester, Manchester, UK; 14 NIHR Clinical Research Clinical Trials, Manchester, UK; 15 Bradford Institute for Health Research, Bradford, UK

**Keywords:** Disease genetics, Cholestasis, Protein function predictions, Risk factors

## Abstract

This study assessed the contribution of five genes previously known to be involved in cholestatic liver disease in British Bangladeshi and Pakistani people. Five genes (*ABCB4*, *ABCB11*, *ATP8B1*, *NR1H4*, *TJP2*) were interrogated by exome sequencing data of 5236 volunteers. Included were non-synonymous or loss of function (LoF) variants with a minor allele frequency < 5%. Variants were filtered, and annotated to perform rare variant burden analysis, protein structure, and modelling analysis *in-silico*. Out of 314 non-synonymous variants, 180 fulfilled the inclusion criteria and were mostly heterozygous unless specified. 90 were novel and of those variants, 22 were considered likely pathogenic and 9 pathogenic. We identified variants in volunteers with gallstone disease (n = 31), intrahepatic cholestasis of pregnancy (ICP, n = 16), cholangiocarcinoma and cirrhosis (n = 2). Fourteen novel LoF variants were identified: 7 frameshift, 5 introduction of premature stop codon and 2 splice acceptor variants. The rare variant burden was significantly increased in *ABCB11*. Protein modelling demonstrated variants that appeared to likely cause significant structural alterations. This study highlights the significant genetic burden contributing to cholestatic liver disease. Novel likely pathogenic and pathogenic variants were identified addressing the underrepresentation of diverse ancestry groups in genomic research.

## Introduction

Cholestatic liver disease encompass a broad range of diagnoses that can present with fatigue, pruritus or jaundice^1^. Several genes are implicated, including the *ABCB4* gene coding for the canalicular phosphatidylcholine floppase ABCB4, and the *ABCB11* gene coding for the bile salt export pump (BSEP). Homozygous mutations in *ABCB4* and *ABCB11* cause a spectrum of disease from mild cholestasis to severe progressive familial intrahepatic cholestasis (PFIC), PFIC3 and PFIC2 respectively^2,3^. *ABCB4* variants also increase the risk of developing drug-induced intrahepatic cholestasis, gallstone disease, gallbladder and bile duct carcinoma, liver cirrhosis and abnormal liver function tests^4^. Other canalicular transporters and their regulators are implicated in the pathogenesis of cholestatic liver disease e.g. *ATP8B1* (a P-type ATPase that flips phospholipids into the cytoplasmic leaflet of the membrane)^3^, *NR1H4* (farnesoid X receptor (FXR)) gene^5^, and *TJP2* (tight junction protein 2)^6^. While homozygous mutations of these genes are implicated in rare cases of severe familial cholestasis, the evidence base for a role of heterozygous mutations in milder forms of liver disease is limited. *ABCB4* and *ABCB11* are involved in 20% of patients with severe intrahepatic cholestasis of pregnancy (ICP). Heterozygous *ABCB4* variants have also been reported in ICP^7–13^. ICP is the commonest gestational liver disease^14^ and may be complicated by preterm birth, meconium-stained amniotic fluid and stillbirth, in association with maternal serum bile acid concentrations ≥ 40 µmol and the timing of its occurrence during pregnancy^15–17^. In Europeans the incidence is 0.62% versus 1.24% in women of Indian and 1.46% of Pakistani origin^18^. Despite the increased prevalence of ICP and other liver conditions like non-alcoholic fatty liver disease in South Asian populations they often remain undiagnosed and under-investigated^19,20^.

Genes and Health is a long-term population-based cohort that assesses the health and disease in British Bangladeshi and British Pakistani people. Using the Genes & Health genomics (whole exome sequencing (WES)) and data linkage to electronic health records (EHR)^21^, this study investigated rare variation in a unique British Bangladeshi and Pakistani cohort around 5 candidate loci (*ABCB4*, *ABCB11*, *ATP8B1*, *NR1H4*, and *TJP2*) implicated in cholestatic liver disease.

## Results

### Genotype to phenotype analysis

Screening of the 5 candidate genes identified 300 variants; and 180 (166 non-synonymous and 14 loss of function (LoF) variants), were included in the analysis (Table 1). Most variants identified were heterozygous unless otherwise specified. A small number of volunteers carried more than one variant, and this is summarised in Supplementary Table 1. None of these volunteers displayed a disease phenotype. Further variant interpretation including scoring details of individual *in-silico* predictors, the impact of the coding substitution on disease propensity and conservation information for all variants are presented in Supplementary Table 2.

**Table 1:** Overall summary of mutational burden discovered in the Genes and Health cohort for all five gene candidates.

Gene	Overall summary of variants (n)				
	ABCB4	ABCB11	ATP8B1	NR1H4	TJP2
NSV	68	77	50	22	83
After inclusion criteria					
Total	41	48	31	9	37
Pathogenicity					
LP	22	25	3	0	0
VUS	13	18	23	8	35
Benign	6	4	5	1	2
Phenotypes					
ICP	5	5	1	2	3
GD	7	10	6		8
Other	1 (cholangiocarcinoma)		1 (cirrhosis), 1 (cirrhosis with secondary malignant neoplasm of liver and bile duct, and gallstone disease)		

Inclusion criteria (< 5% MAF), NSV and at least any of the following: 1. Include all variants with a phenotype 2. Include all variants known in the literature 3. Include all variants with no GnomAD allele frequency but ELGH allele frequency 4. Include all variants with in silico prediction of 7.

*GD*, gallstone disease, *ICP* intrahepatic cholestasis of pregnancy, *LP* likely pathogenic, *MAF* minor allele frequency, *NSV* non-synonymous variants, *VUS* variant of unknown significance.

### Phenotype to genotype analysis

We were able to validate variants of interest in 15 cases reporting ICP, most of whom had documented raised BA concentrations (Table 2). Further, variants discovered in 10 cases with raised BA but an uncertain or no diagnosis of ICP based on their EHR (Supplementary Table 3). This secondary analysis missed 2 individuals from the primary analysis with a diagnosis of ICP as there were no bile acid concentrations available for them. This analysis demonstrated a pragmatic approach to identifying disease causing variants and demonstrates the value of large genomic cohorts linked to electronic health data records.

**Table 2:** Variants identified and TSBA concentration in volunteers with a diagnosis of ICP based on electronic health records.

Volunteers	All variants in volunteers with raised BA and diagnosis of ICP				Highest BA (umol/L)
	Gene	Variants	Zygosity	Type	
3	ABCB4	G1254S/G1261S	het	Non-synonymous	80.1
3	ABCB11	N591S	het	Non-synonymous	
4	ABCB4	P1050S	het	Non-synonymous	53
5	TJP2	Q105K	hom	Non-synonymous	15
6	ABCB11	N591S	het	Non-synonymous	17
13	TJP2	Q105K	het	Non-synonymous	25
15	ATP8B1	R384H	het	Non-synonymous	31.6
15	TJP2	Q105K	hom	Non-synonymous	
18	ABCB11	N591S	het	Non-synonymous	115
18	TJP2	R21H	het	Non-synonymous	
20	ABCB11	N591S	het	Non-synonymous	18
20	NH1R4	M173T	het	Non-synonymous	
21	ABCB11	M677V	het	Non-synonymous	14
21	NH1R4	N358H	het	Non-synonymous	
22	ABCB11	N591S	het	Non-synonymous	14
25	ABCB4	S99x	het	Frameshift	55
26	ABCB11	V284A	het	Non-synonymous	35
26	ABCB4	N510S	het	Non-synonymous	
31	ABCB4	T175A	het	Non-synonymous	32
32	ABCB11	D1284N	het	Non-synonymous	
32	ABCB11	N591S	het	Non-synonymous	
38	ABCB11	N591S	het	Non-synonymous	45.2

*TSBA* total serum bile acid concentrations, *ICP* intrahepatic cholestasis of pregnancy.

### Rare variant burden analysis

We observed in cases versus controls a significant enrichment of rare variants in *ABCB11* (p = 0.00247). There was no enrichment in *ABCB4* (p = 0.39138), *ATP8B1* (p = 0.57957)*, TJP2* (p = 0.17390)*,* or *NR1H4* (p = 0.70232)*.* A further Fisher’s exact analysis compared percentage of rare variants in ICP cases in Genes & Health compared to Genomics England demonstrating that the rare genetic burden was significantly increased in tier 1 gene candidates in British Pakistani and Bangladeshi (*ABCB4*, p = 0.0191; *ABCB11*, p = 0.0191) but not in *ATP8B1* (p = 0.4857) *NR1H4* (p = 0.2286) or *TJP2* (p = 0.1039).

### ABCB4 variants

**Figure 1:**
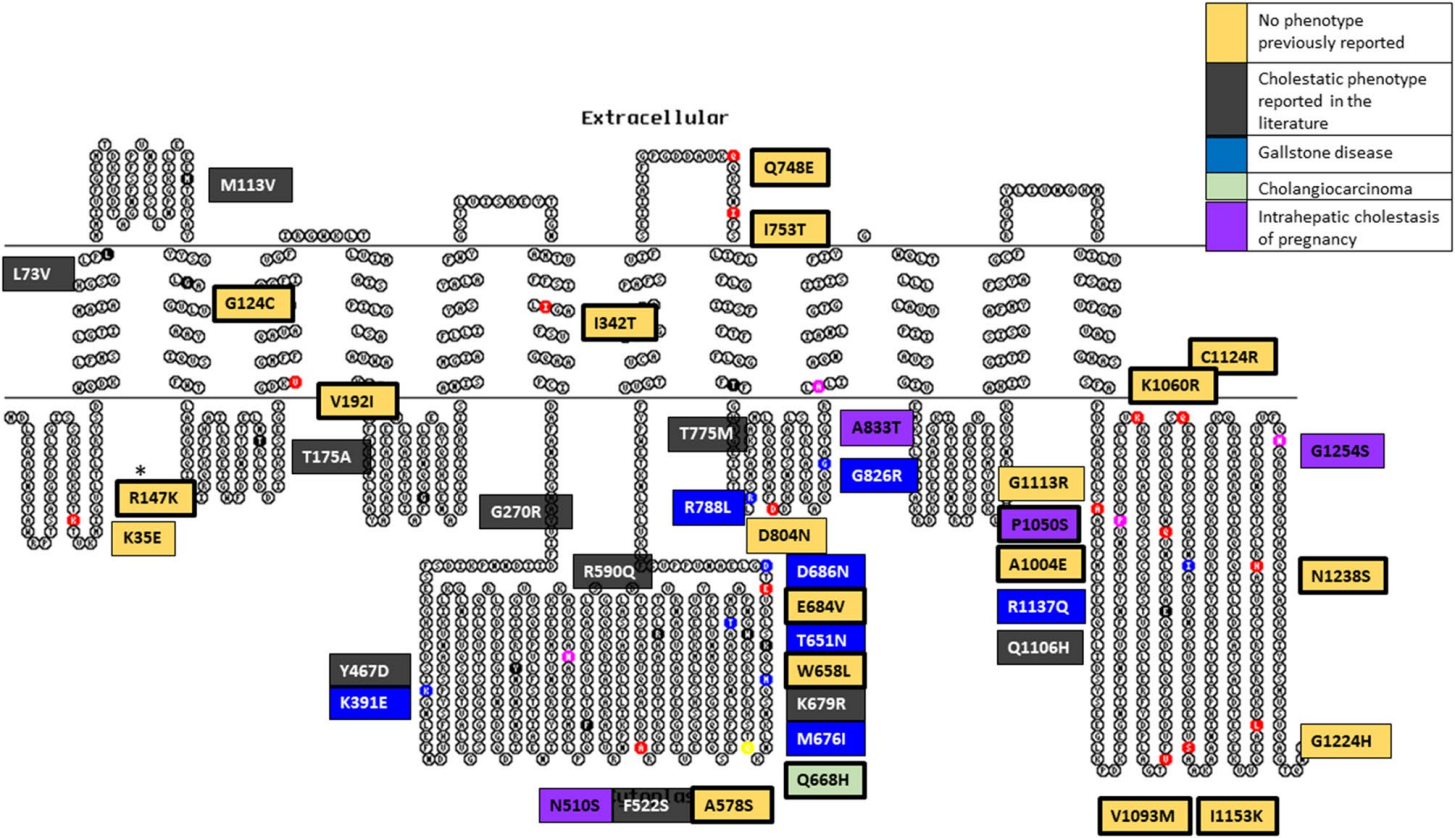
ABCB4 variant summary in a 2-dimensional illustration. 41 variants are divided into their phenotypic presentation and coloured by: No phenotype previously reported (n = 19), cholestatic phenotype reported in the literature (n = 9), gallstone disease (n = 7), cholangiocarcinoma (n = 1), and intrahepatic cholestasis of pregnancy (n = 5). Bold border represents variants that are unique to the Genes & Health cohort. Topo2 software (Johns S.J., TOPO2, Transmembrane protein display software, http://www.sacs.ucsf.edu/TOPO2) was used for illustration^95^.

There was a total of 68 *ABCB4* variants identified (Table 1 and Fig. 1). 41 variants fulfilled the inclusion criteria. Variants were identified in people with cholestatic liver disease phenotypes: ICP (n = 5 out of 88 women in the analysis), gallstone disease (n = 7), and cholangiocarcinoma (n = 1) (Table 3). For some identified variants a known cholestatic phenotype had previously been reported in the literature (n = 9) whilst others had no phenotype reported (n = 19) (Supplementary Table 4). We identified novel LoF variants (n = 4): three frameshift (one associated with an ICP phenotype) and one introduction of a premature stop codon (no associated phenotype) (Table 4).

**Table 3:** Non-synonymous variants identified in the five gene candidates associated with a cholestatic phenotype in the Genes and Health cohort.

Gene	Phenotype	Transcript	Protein change	dbSNP	gnomAD AF	G&H AF^	ACMG-AMP	ACMG-AMP criteria	Clinvar	Ref
ABCB4	Intrahepatic cholestasis of pregnancy	ENSP00000496956.1:p.Gly1254Ser	G1254S*	rs781315185	0.00003656	0.00028843	LP	PM1, PM2, PP2, PP3		^22^
		ENSP00000497931.1:p.Asp1284Asn	P1050S*			0.00019146	LP	PM1, PM2, PP2, PP3		
		ENSP00000496956.1:p.Ala833Thr	A833T*			0.00009638	LP	PM1, PM2, PP2, PP3		
		ENSP00000496956.1:p.Asn510Ser	N510S	rs375315619	0.00019110	0.00057394	LP	PM1, PM2, PP2, PP3, PP5	LP	^23–30^
		ENSP00000496956.1:p.Thr175Ala	T175A^1^	rs58238559	0.01155000	0.01315030	LB	PM1, PP2, PP3, BS1, BS2, BP6	Benign/likely benign	^3,12,23,25,26,28–43^
	Gallstone disease	ENSP00000496956.1:p.Arg1137Gln	R1137Q	rs780738927	0.00003250	0.00028846	LP	PM1, PM2, PP2, PP3		
		ENSP00000496956.1:p.Gly826Arg	G826R*			0.00028681	LP	PM1, PM2, PP2, PP3		
		ENSP00000496956.1:p.Arg788Leu	R788L	rs8187801	0.00000813	0.00009579	LP	PM1, PM2, PP2, PP3	Benign	
		ENSP00000496956.1:p.Asp686Asn	D686N	rs78653500		0.00009586	VUS	PM2, PP2, BP4		
		ENSP00000496956.1:p.Met676Ile	M676I	rs376702091	0.00002033	0.00038278	VUS	PM2, PP2, BP4		
		ENSP00000496956.1:p.Thr651Asn	T651N	rs45476795	0.0005776	0.0006719	LB	PM2, PP2, BP4, BP6	conflicting	
		ENSP00000496956.1:p.Lys391Glu	K391E	rs781347049	0.00002440	0.00009582	LB	PM2, PP2, BP4, BP6		
	Cholangiocarcinoma	ENSP00000496956.1:p.Gln668His	Q668H*			0.00009586	LB	PM2, PP2, BP4		
ABCB11	Intrahepatic cholestasis of pregnancy	ENSP00000497931.1:p.Asp1284Asn	D1284N	rs766784155	0.00001228	0.00028780	LP	PM1, PM2, PP2, PP3		
		ENSP00000497931.1:p.Arg1050His	R1050H	rs72549398	0.00000421	0.00019135	VUS	PM2, PP2, BP4		
		ENSP00000497931.1:p.Met677Val	M677V^2^	rs11568364	0.02364000	0.01005750	Benign	PP2, BA1, BS3, BP6, BP4	Benign	^12,43–52^
		ENSP00000497931.1:p.Asn591Ser	N591S^3^	rs11568367	0.01436000	0.12647200	Benign	PM1, PP2, BA1, BP6	Benign	^3,12,28,36,46,52–61^
		ENSP00000497931.1:p.Val284Ala	V284A	rs200739891	0.00026040	0.00009558	LP	PM1, PM2, PM5, PP2, PP3, BP6	Conflicting	^31,48,50^
	Gallstone disease	ENSP00000497931.1:p.Ala1260Pro	A1260P	rs772097949	0.00001641	0.00028153	LP	PM1, PM2, PP2, PP3	VUS	
		ENSP00000497931.1:p.Gln976Arg	Q976R	rs199940188	0.00054840	0.00066883	VUS	PM1, PM2, PP2, BP4	Conflicting	
		ENSP00000497931.1:p.Ala926Ser	A926S*			0.00040667	LP	PM1, PM2, PM5, PP2,PP3		
		ENSP00000497931.1:p.Ala679Val	A679V	rs200912109	0.00045560	0.00143761	VUS	PM2, PP2, BP4	Conflicting	
		ENSP00000497931.1:p.Asn539Asp	N539D*			0.00022604	VUS	PM1, PM2, PP2, BP4		
		ENSP00000497931.1:p.Arg487Cys	R487C	rs770693935	0.00002043	0.00009549	LP	PM1, PM2, PP2, PP3		^62^
		ENSP00000497931.1:p.Ala311Thr	A311T	rs200509511	0.00004073	0.00028969	LP	PM1, PM2, PP2, PP3		
		ENSP00000497931.1:p.Val95Ile	V95I	rs201735739	0.00009766	0.00028708	Benign	PM1, PM2, PP2, BP4		
		ENSP00000497931.1:p.Asp94Asn	D94N	rs760920706	0.00010170	0.00095621	LP	PM1, PM2, PP2	Conflicting	
		ENSP00000497931.1:p.Lys12Arg	K12R*			0.00010378	LP	PM1, PM2, PP2, PP3		
ATP8B1	Intrahepatic cholestasis of pregnancy	ENSP00000497896.1:p.Arg384His	R384H#	rs2271260	0.00026400	0.00048040	VUS	PM2, PP2, BP6		^3^
	Gallstone disease									
	Gallstone disease	ENSP00000497896.1:p.Val1161Ala	V1161A	rs1255793857	0.00000406	0.00009549	VUS	PM2, PP2		
		ENSP00000497896.1:p.Thr1092Ile	T1092I	rs780425796	0.00001220	0.00030581	VUS	PM2, PP2, PP3		
		ENSP00000497896.1:p.Met674Thr	M674T+^4^	rs35470719	0.00456300	0.00632063	Benign	PP2, BA1, BP4, BP6	Benign/Likely benign	^52,63–68^
		ENSP00000497896.1:p.Ile577Val	I577V+^5^	rs3745078	0.00467800	0.00628992	Benign	PP2, BA1, BP6	Benign	^52,63–66,68^
		ENSP00000497896.1:p.His78Gln	H78Q+^6^	rs3745079	0.00421800	0.00495751	Benign	PP2, BP4,BP6, BS1, BS2	Benign	^52,63–66,68^
		ENSP00000497896.1:p.Asp14Tyr	D14Y*			0.00009560	VUS	PM1, PM2, PP2, BP4		
	Cirrhosis	ENSP00000497896.1:p.Ile513Thr	I513T	rs772028343	0.00008531	0.00066973	VUS	PM2, PP2		
	Cirrhosis, secondary malignant neoplasm of liver and bile duct, gallstone disease	ENSP00000497896.1:p.Asp70Asn	D70N^7^	rs34719006	0.00313900	0.00302678	VUS	PM2, PP2, PP3	Conflicting	^24,64,69–75^
NR1H4	Intrahepatic cholestasis of pregnancy	ENSP00000496908.1:p.Asn358His	N358H	rs149287629	0.00041020	0.00038307	VUS	PM2	VUS	
		ENSP00000496908.1:p.Met173Thr	M173T	rs61755050	0.00374800	0.00267482	Likely benign	PM1, PP3, BS1, BS2, BP6	likely benign	^6,76^
TJP2	Intrahepatic cholestasis of pregnancy	ENSP00000497787.1:p.Thr377Ala	T377A	rs766748789	0.00000406	0.00047765	VUS	PM2, BP4		
		ENSP00000496791.1:p.Gln105Lys	Q105K^8^	rs41305539	0.05150000	0.12031400	Benign	BA1, BP4, BP6,	benign	^44,77,78^
		ENSP00000497861.1:p.Arg21His	R21H^9^	rs4493966	0.07416000	0.04555170	Benign	BA1, BP4, BP6,	benign	
	Gallstone disease	ENSP00000496791.1:p.Gln8Arg	Q8R*			0.00009553	VUS	PM2, PP3		
		ENSP00000496791.1:p.Thr68Asn	T68N*			0.00019150	VUS	PM1, PM2		
		ENSP00000496791.1:p.Pro152Leu	P152L	rs754300892	0.00007876	0.00046685	VUS	BP4		
		ENSP00000497787.1:p.Arg178Cys	R178C	rs199761505	0.00043060	0.00223305	VUS	PP3		
		ENSP00000497787.1:p.Arg255His	R255H	rs532438219	0.00012990	0.00066947	VUS			
		ENSP00000497787.1:p.Arg461Pro	R461P	rs748523814	0.00009746	0.00078125	VUS	PP3		
		ENSP00000496791.1:p.Thr902Met	T902M	rs774198938	0.00010970	0.00010449	VUS	PP3		
		ENSP00000496791.1:p.Arg1070Lys	R1070K*			0.00009564	VUS	PM2, BP4	VUS	

*AF* allele frequency, *ACMG-AMP* American College of Medical Genetics and Genomics and the Association for Molecular Pathology, *BP* benign supporting, *PM* pathogenic moderate, *PP* pathogenic supporting, *G&H* Genes & Health, *LP* likely pathogenic, *Ref* references, *VUS* variant of unknown significance.

*South Asian specific variant.

#multiple phenotype.

+Linkage disequilibrium.

^Allele frequency specific to East London Genes & Health cohort.

^1^T175A, Hom (n) 6, Het (n) 126.

^2^M677V, Hom (n) 3, Het (n) 99.

^3^N591S, Hom (n) 99, Het (n) 760.

^4^M674T, Hom (n) 2, Het (n) 67.

^5^I577V, Hom (n) 2, Het (n) 61.

^6^H78Q, Hom (n) 1, Het (n) 47.

^7^D70N, Hom (n) 1, Het (n) 24.

^8^Q105K, Hom (n) 76, Het (n) 711.

^9^R21H, Hom (n) 16, Het (n) 443.

**Table 4:** Loss of function variants identified in the five gene candidates.

Phenotype	Gene	Transcript	Protein change	Info	gnomAD AF	G&H AF^	ACMG-AMP	ACMG-AMP criteria	Clinvar
ICP	ABCB4	ENSP00000395716.1:p.Ser99LeufsTer11	S99x	Frameshift	0.00039970	0.00336538	P	PVS1, PM2, PP3	
		ENSP00000392983.1:p.Leu759TyrfsTer38	F758x	Frameshift		0.00009610	LP	PVS1, PM2	
		ENSP00000392983.1:p.Lys30GlyfsTer7	Lys30Glyfster7	Frameshift	0.00000408	0.00020243	LP	PVS1, PM2	
		ENSP00000392983.1:p.Arg595Ter	R595*	Stop-gained	0.00001627	0.00009593	P	PVS1, PM2, PP3, PP5	Pathogenic
Gallstone disease	ABCB11	ENSP00000497931.1:p.Ala1044LeufsTer53	A1044x	Frameshift		0.00009562	P	PVS1, PM2, PP3	
		ENST00000263817.7:c.2611-2A > T	c.2611-2A > T	Splice-acceptor-variant	0.00000407	0.00057870	P	PVS1, PM2, PP3	
		ENSP00000497931.1:p.Trp239Ter	W239x	Stop-gained		0.00009566	P	PVS1, PM2, PP3	
	ATP8B1	ENSP00000283684.4:p.Gln1179GlufsTer56	IQ1178-1179IX	Frameshift_variant & splice_region_variant		0.00193798			
		ENSP00000283684.4:p.Pro792HisfsTer8	F791X	frameshift_variant		0.00019069	P	PVS1, PM2, PP3	
Gallstone disease		ENST00000283684.9:c.182-4_183del	?-61	Splice_acceptor_variant & coding_sequence_variant & intron_variant		0.00048956			
		ENSP00000283684.4:p.Glu20Ter	E20*	Stop_gained		0.00009566	P	PVS1, PM2, PP3	
	NR1H4	ENSP00000446760.1:p.Lys4Ter	K4*	Stop_gained		0.00014188	P	PVS1, PM2, PP3	
	TJP2	ENSP00000438262.1:p.Glu44Ter	E44*	Stop_gained		0.00009604	LP	PVS1, PM2	
		ENSP00000345893.4:p.Gly5ArgfsTer26	M1MPVX	Frameshift_variant & start_lost	0.00001343	0.00029768	P	PVS1, PM2, PP3	

*AF* allele frequency, *ACMG-AMP* American College of Medical Genetics and Genomics and the Association for Molecular Pathology, *BP* benign supporting, *PM* pathogenic moderate, *PP* pathogenic supporting, *G&H* Genes & Health, *LP* likely pathogenic, *P* pathogenic.

^Allele frequency specific to East London Genes & Health cohort.

### ABCB11 variants

**Figure 2:**
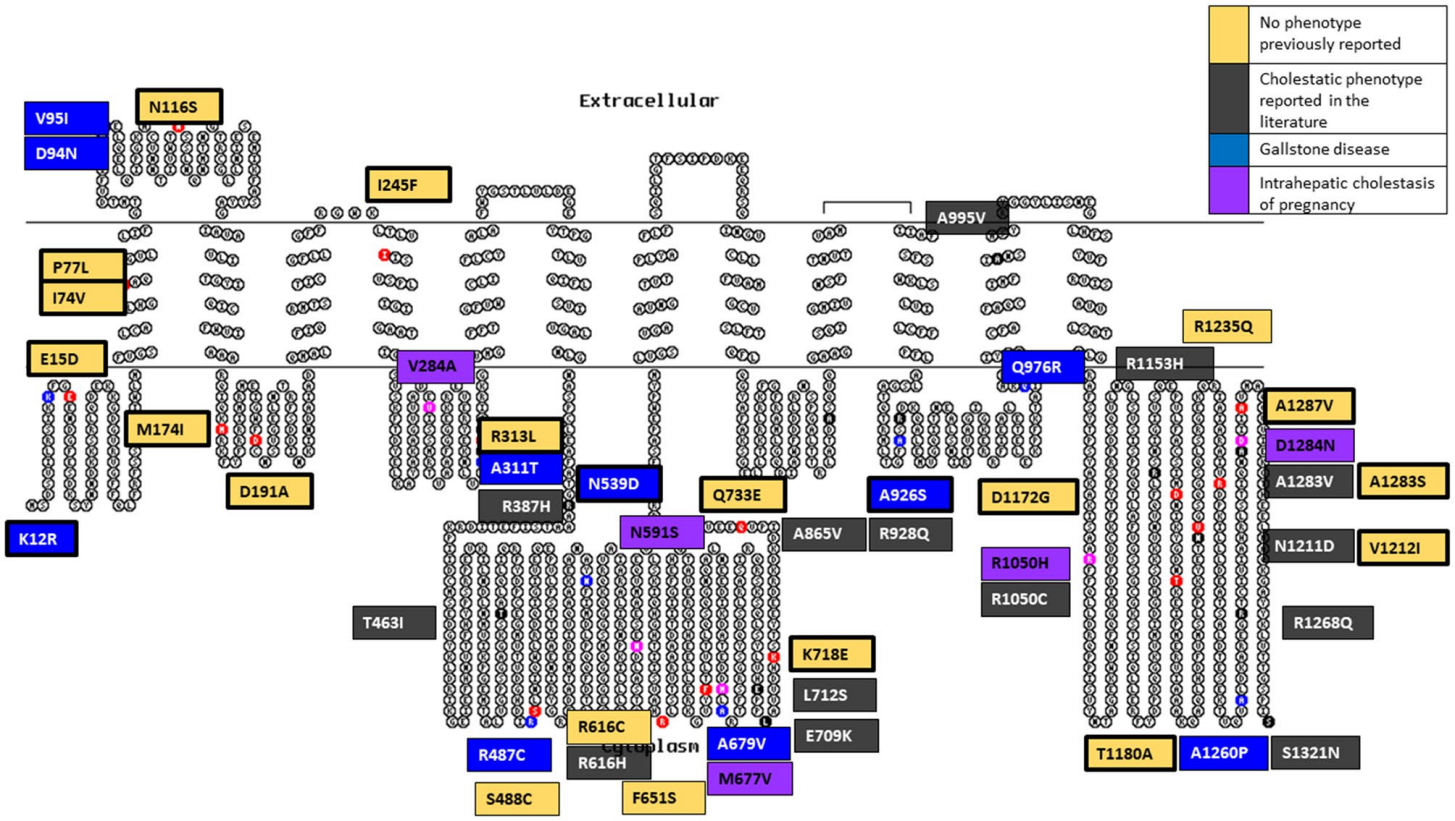
ABCB11 variant summary in a 2-dimensional illustration. 48 variants are divided into their phenotypic presentation and coloured by: No phenotype previously reported (n = 19), cholestatic phenotype reported in the literature (n = 14), gallstone disease (n = 10), and intrahepatic cholestasis of pregnancy (n = 5). Bold border represents variants that are unique to the Genes & Health cohort. Topo2 software (Johns S.J., TOPO2, Transmembrane protein display software, http://www.sacs.ucsf.edu/TOPO2) was used for illustration^95^.

There were 77 *ABCB11* variants identified of which 48 were included in the analysis (Table 1 and Fig. 2). The associated cholestatic liver disease phenotypes identified were: ICP (n = 5 out of 83 women in the analysis), and gallstone disease (n = 10) (Table 3). Some were linked to cholestatic phenotypes previously reported in the literature (n = 14), whilst for 19, no phenotype had been previously reported (n = 19) (Supplementary Table 5). Likely novel LoF variants were identified (n = 3): a frameshift, a splice-acceptor and an introduction of premature stop codon variant. These variants were not associated with a known disease phenotype (Table 4).

### ATP8B1 variants

**Figure 3:**
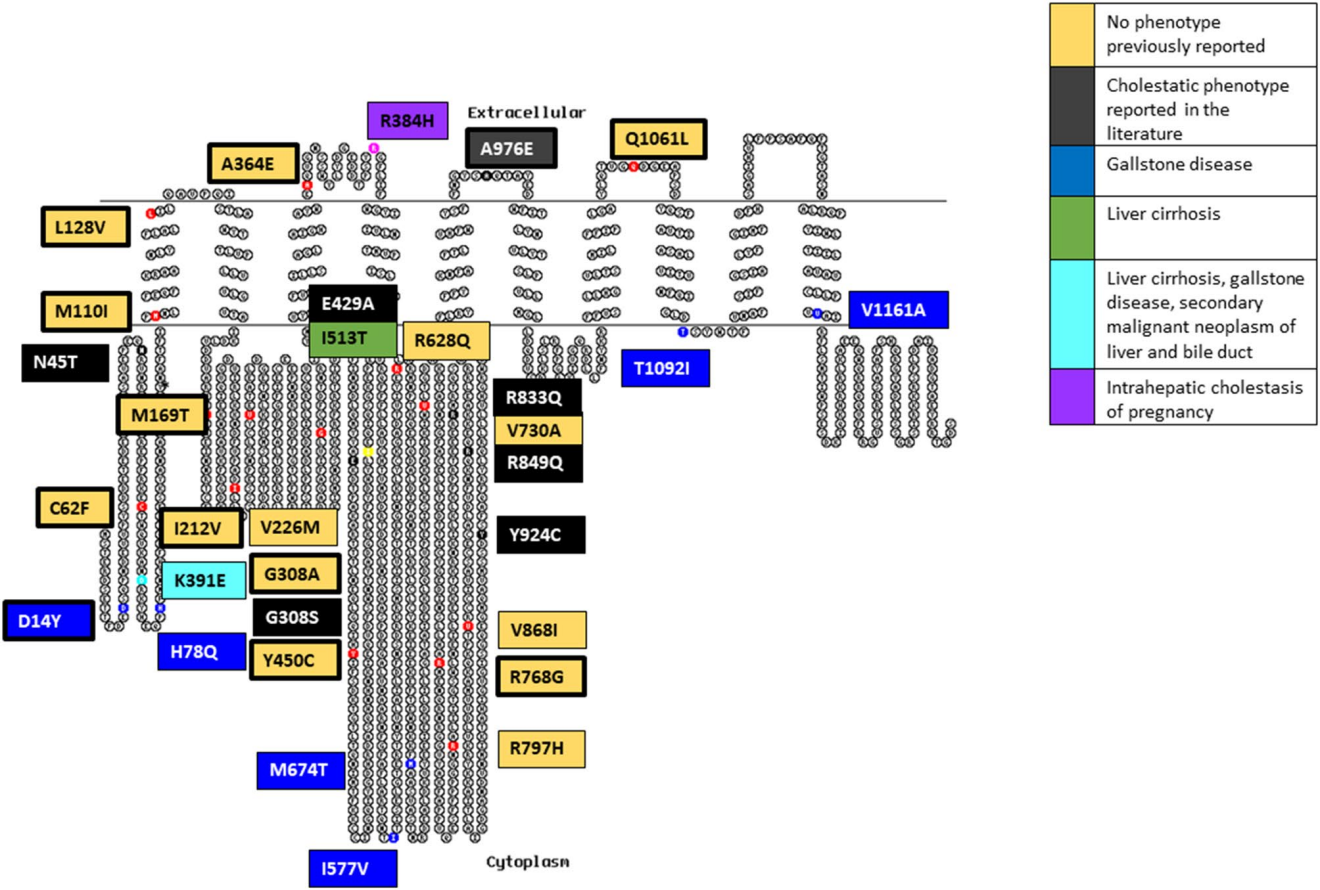
ATP8B1 variant summary in a 2-dimensional illustration. 31 variants are divided into their phenotypic presentation and coloured by: No phenotype previously reported (n = 15), cholestatic phenotype reported in the literature (n = 7), gallstone disease (n = 7), liver cirrhosis (n = 1), Liver cirrhosis and multiple cholestatic phenotype (n = 1), and intrahepatic cholestasis of pregnancy (n = 2). Bold border represents variants that are unique to the Genes & Health cohort. Topo2 software (Johns S.J., TOPO2, Transmembrane protein display software, http://www.sacs.ucsf.edu/TOPO2) was used for illustration^95^.

We identified a total of 50 *ATP8B1* variants and 31 were included in the final analysis (Table 1 and Fig. 3). There were 7 variants associated with gallstone disease; three appeared to be in linkage disequilibrium (LD) noted in three volunteers: M674T, I577V, and H78Q. The R384H variant was associated with gallstone disease and with an ICP phenotype (in separate individuals, n = 2 out of 33 women in the analysis). A further variant was associated with hepatitis-induced liver cirrhosis (I513T), and a final variant (D70N) was associated with liver cirrhosis, secondary malignant neoplasm of liver and bile duct, and gallstone disease (Table 3). In addition, previously reported cholestatic liver disease phenotypes (n = 7) and variants with no previous reported phenotype were seen (n = 15) (Supplementary Table 6). Finally, we identified 4 novel LoF variants: a frameshift/splice region, frameshift, splice-acceptor/coding-sequence, and stop-gain variant. The latter variant was associated with a gallstone disease phenotype (Table 4).

### NR1H4 variants

There were 22 *NR1H4* variants in the Genes & Health cohort and 9 variants in the final analysis (Table 1 and Supplementary Fig. 2). We only identified an ICP phenotype (n = 2 out of 33 women in the analysis) (Table 3) and otherwise novel variants that had no previous phenotype reported (n = 7) (Supplementary Table 7). Furthermore, one novel LoF variant was identified without demonstrating a phenotype (Table 4).

### TJP2 variants

There were 83 *TJP2* variants identified of which 37 were analysed (Table 1). People with *TJP2* variants had ICP (n = 3 out of 103 women in the analysis), gallstone disease (n = 8), previously reported cholestatic liver disease phenotype (n = 4), and 22 did not have a previously reported phenotype (Table 3, Supplementary Table 8). There were two novel LoF variants without a clinical phenotype (Table 4).

### Protein structure and molecular modelling

A flow chart illustrating the variants included in this analysis is shown in Supplementary Fig. 3. Results of the protein structure and molecular modelling software tools are presented in Supplementary Table 9.

Some novel variants are in regions of transporters for which we can hypothesis a mechanistic impact. Of particular interest are Q1106H in ABCB4 and D191A in ABCB11. These ABC B-family transporters share 48% amino acid identity and are very likely have a common mechanism of action. The two amino acids are conserved in both proteins, and we propose that they are involved in energy transduction through the transporter in order to couple the substrate efflux cycle to the ATP binding and catalysis cycle.

In ABCB4 and ABCB11, two transmembrane domains (TMDs) bind the transport substrates (phosphatidylcholine (PC) and bile acids), respectively. The conformational changes required for substrate transport are driven by ATP hydrolysis at the interface between two nucleotide binding domains (NBDs). The TMDs and NBDs must therefore be intimately coupled, and this is achieved via four ‘coupling helices’ (CH) located at the base of the long intracellular loops extending from the transmembrane alpha helices of the TMDs (Supplementary Fig. 4A).

Q1106 (ABCB4) and D191 (ABCB11) are particularly interesting because they are located at this interface that is conserved in both ABCB4 and ABCB11. Q1106 is in a groove on the surface of NBD2 where it interacts with CH2 (Supplementary Fig. 4B).

In the PC-bound conformation of ABCB4, Q1106 forms a weak electrostatic interaction with the peptide bond of G270. In the ATP-bound conformation (from which PC has most likely been released), Q1106 now interacts with Q272 which illustrates the movement of CH2 and its changing juxtaposition with the NBD during the transport cycle; essentially, a hinge region. The geometry of these interactions will not be preserved if Q1106 is replaced by histidine. In ABCB11, this triad is preserved in Q1150 and G295, with E297 providing a conservative change for the glutamine in CH2 (with respect to formation of an equivalent electrostatic bond with Q1150).

In the sole structural model that we have for ABCB11, D191 is in CH1 where it interacts with Y472 in a surface groove of NBD1 and also, intriguingly, with R946 which is in CH4, suggesting that CH1 and CH4 likely work together in energy transduction through the transporter (Supplementary Fig. 4C).

These electrostatic bonds will not be possible if D191 is replaced with alanine. This triad and its bond architecture is perfectly conserved in ABCB4 in the ATP bound conformation through amino acids D166, Y446 and R902. However, in ABCB4 there is also an additional electrostatic interaction between the carbonyl of the D166 peptide bond and the side chain of Q1171. Q1171 (which is conserved in ABCB11) is adjacent to the ABC signature motif ^1172^LSGGQ^1176^ which is involved in coordination of ATP and provides a direct mechanism for how CH1 influences, and responds to, the ATP catalytic cycle of these transporters.

## Discussion

This study identified novel variants implicated in the aetiopathogenesis of cholestatic liver disease that occur uniquely in this British Bangladeshi and Pakistani cohort^36,79–81^. There have not been any other studies of this magnitude analysing the burden of mutational variation in cholestatic liver disease in a large South Asian cohort. Using a genotype to phenotype approach we discovered novel likely pathogenic variants that appear to be unique to this cohort. We then employed a phenotype to genotype analysis using the ICP phenotype as an exemplar, which offered a pragmatic interrogation of electronic health records to identify rare genetic variants that are likely pathogenic. Thus, this study improves representation of this distinct population especially as prevalence of cholestatic liver disease is increased in the Genes & Health cohort, e.g. 1.54% are affected by ICP compared to white Europeans (0.62%). This study demonstrates the importance of multi-ancestry genomic research and offers the potential of tailored treatment for this population.

In the Genes & Health cohort, out of 194 variants meeting inclusion criteria we identified 53 that had a cholestatic liver disease phenotype reported in their linked EHR. Of those, 16 are unique to this British Bangladeshi and Pakistani population and a large number were predicted to be likely pathogenic or known pathogenic based on *in-silico* prediction tools. In addition, there were 35 variants that were previously reported in the literature with a cholestatic phenotype. However, 87 variants had no previously reported phenotype; 67 were novel (34% of all variants analysed in this study) as they were also not previously reported in the GnomAD population database. Despite that, 9 were considered likely pathologic and 5 known pathogenic. It is important to consider that heterozygosity as noted in most cases means that they are likely rescued by the wild-type allele but at higher risk of disease in later life or during times of liver stress, e.g. during pregnancy.

Our findings reflect the difficulty with interpretation of rare variants in clinically important genes when there is no previous evidence in the literature or functional data to interpret them further. The ACMG rare variant interpretation guideline^30^ provides a standardised analysis pathway. However, it relies in part on the interpretation of the variant in the context of the literature and does not account for specific genes and diseases. It also may not be robust for flexible membrane proteins which do not work by lock and key mechanism. For example, the *ABCB11* variant V444A, considered as benign by the ACMG criteria, has been reported to increase the risk of ICP, hepatitis C disease progression, and drug-induced liver injury although the exact functional mechanisms are not clear yet^55,82^.

By employing computational protein modelling software tools, we were able to identify variants that likely have a significant impact on the conformation of the protein and could therefore be of clinical significance. It is important to bear in mind that all these tools have inherent flaws and are beyond the scope of this paper to discuss in detail. By taking ICP as a cholestatic liver disease example we were able to highlight further difficulties with rare variant interpretation in gestational syndromes as the inherent transient nature of the disease makes variant interpretation challenging. However, ICP is a clinically relevant example as the gestational disease consequences are not just relevant to their current pregnancy but also can result in later hepatobiliary disorders such as cancer, immune-mediated and cardiovascular diseases^83^. In addition, they have a higher gallstone-related morbidity and a strong positive association between ICP and hepatitis C exists as well^84^.

## Limitations

The use of electronic health records to determine phenotype is extremely useful but dependent upon appropriate information having been coded. Participants with at-risk variants may not have presented yet with symptoms of disease but still be at high risk of developing complications at a later stage in their life, particularly given that the median age of volunteers in this study was around 45 years. It demonstrates the difficulty with interpreting variants when recalling the genotype first.

## Conclusions

In this study we provide the first comprehensive evaluation of gene candidates associated with cholestatic liver diseases in a unique cohort of British Bangladeshi and Pakistani origin demonstrating the importance of characterising genetic disease in diverse ethnic groups. Our findings have demonstrated the increased mutational burden of cholestatic liver disease in British Bangladeshi and Pakistani people who thus far remain understudied despite their distinct genetic background and increased risk of developing ICP in comparison to other populations. We were able to identify novel variants that have not been previously identified and are likely implicated in disease. We demonstrated the ability to identify participants at risk both by a phenotype or genotype first approach. This demonstrates the importance of providing more personalised care in a clinical setting as identification of high-risk individuals and their family members enables early intervention to prevent adverse outcomes, for example hepato-protective drugs such as UDCA, in addition to hepatic surveillance. Furthermore, it provides the necessary foundation for improved therapy and drug development.

## Methods

### Study population

A detailed description of the Genes & Health cohort has been described by Finer et al.^21^. Ethical approval for the study was provided by the South East London National Research Ethics Committee (14/LO/1240) including consent for publishing http://www.genesandhealth.org/volunteer-information^22^. All Genes & Health volunteers consented to lifelong EHR linkage, DNA extraction and genetic tests. All research was conducted in accordance with NHS Health Research Authority guidelines and regulations. An individual application to support data access for this study was granted by Genes & Health (reference S00037) taking into consideration community prioritisation, acceptability and scientific merit.

Exome sequencing samples from 5236 Genes & Health volunteers reporting parental relatedness were available for analysis in variant call format files. For the initial analysis a genotype to phenotype approach was employed interrogating 5 gene candidate loci (Table 5). For the rare burden analysis female volunteers without ICP served as controls (n = 3048). In a secondary analysis, a phenotype to genotype analysis was used to validate these findings, using ICP as the exemplar. For this approach, electronic health records allowed total serum bile acid concentrations ≥ 10 µmol to be retrieved from a network of acute hospitals that provide maternity care to (n = 15,500) women per year living in east London to identify patients with a diagnosis of liver disease in pregnancy (ICD 10 diagnosis code O26.6), see Supplementary Fig. 1. Maternal health records were screened by an experienced clinician to verify a diagnosis of ICP.

**Table 5:** Description of the five gene candidates.

Genes	Chr	Gene product	Omim ID	Exons	Length	Associated disease
ABCB4	7 (7q21.1)		171,060	27	81 kb	PFIC-3
ABCB11	2 (2q31.1)	BSEP	603,201	28	115 kb	PFIC-2
ATP8B1	18 (18q21.31)	FIC1	602,397	28	157 kb	PFIC-1
TJP2	9 (9q21.11)		607,709	25	153 kb	PFIC-4
NR1H4	12 (12q23.1)	FXR	603,826	11	90 kb	PFIC-5

*Chr* chromosomes.

### Exome sequencing & bioinformatic pipeline

Low/mid exome sequencing was performed as previously described^85^. The exome sequencing data is being held under a data access agreement at the European Genotype-phenome Archive (www.ebi.ac.uk/ega) under accession numbers EGAD00001005469. Minor allele frequency (MAF) was set at < 5% to include rare and low-frequency genetic variants to allow for a comprehensive evaluation.

### Variant annotation

All protein-altering missense, non-sense, frameshift indels or splice site variants identified in the candidate gene set underwent the same processing as described below. Synonymous variants were excluded from further analysis. Variants were filtered and annotated if they met any of the following inclusion criteria (MAF < 5%): 1. associated with a phenotype; 2. known in the literature; 3. no recorded GnomAD allele frequency; 4. predicted to be likely pathogenic (LP) based on all 7 *in-silico* predictors. To assess the likelihood of functional impact of variants a variety of *in-silico* tools were employed, including Polyphen^86^, SIFT^87^, CADD^88^, Revel^89^, MetaLR^90^, MetaSVM^90^ and M_CAP^91^. Open-source databases (Leiden Open Variation Database—LoVD, and ClinVar) including a commercial database (Mastermind) were interrogated to assess whether variants were reported previously in the literature. The American College of Medical Genetics/Association for Molecular Pathology (ACMG/AMP) guidelines for rare variant interpretation was used to assess variant pathogenicity. The guidelines consider a variant to be LP if there is a > 90% certainty it being disease-causing, but below a higher “pathogenic” threshold^92^.

### Rare variant burden analysis

To assess the significance of any rare variant burden of SNPs in all 5 gene candidates the exactCMC function in RVTESTS^93^ was used. The burden was calculated as the proportion of all ICP cases versus control in the Genes & Health cohort and who had at least one alternate allele. Variants with an allele frequency of 0.01 or less were included. ICP cases (n = 18) from the Genomics England database (Project ID 747)—a predominantly European genetic cohort—were accessed to serve as a direct comparison to rare variant burden in Genes & Health.

### Protein structure and modelling analysis

Inclusion criteria for further protein structure and modelling analysis required variants to have an associated phenotype, and/or be predicted to be known pathogenic based on all 7 *in-silico* tools. VarMap was used to assess protein sequence variants^94^. 2D representations were designed using the open source tool TOPO2^95^. Variants that were LP or associated with a phenotype underwent further analysis using: (1) Dynamut^96^ (2) CUPSAT^97^ (3) SNPMuSiC^98^. 3D structural representations were generated using PyMOL software (The PyMOL Molecular Graphics System, Version 2.0 Schrödinger, LLC.) using *ABCB4* with phosphatidylcholine (PDB ID: 7NIV)^99^, *ABCB11* open structure (PDB ID: 6LR0)^100^, *ATP8B1* (PDB ID: 7PY4)^101^, and *NR1H4* (PDB ID: 1OSH)^102^. There is no 3D protein structure available for *TJP2*.

## Supplementary Information

Supplementary Information.

## Data Availability

The imputed genotype data from Genes & Heath are available on EGA (www.ega-archive. org; study accession number: EGAS00001005373). The individual-level phenotypic data will be made available to researchers by completing an application to Genes & Health, following their open access policy described on https://www.genesandhealth.org/research/scientists-using-genes-health-scientific-research.
