# Supplementary material for: Rare variant contribution to cholestatic liver disease in a South Asian population in the United Kingdom
Source: Sci Rep. 2023 May 19;13:8120. doi: 10.1038/s41598-023-33391-w (PMC10199085; doi:10.1038/s41598-023-33391-w)
Supplement: Supplementary file 1 — Supplementary Information. [file 41598_2023_33391_MOESM1_ESM.docx]

**Rare variant contribution to cholestatic liver disease in a South Asian population in the United Kingdom**

Julia Zöllner^1^, MBBS, PhD; Sarah Finer^2^, MBBS, PhD; Kenneth J. Linton^3^, PhD; Genes and Health Research Team^4^; David A. van Heel^4^, BM BCh MA, DPhil; Catherine Williamson^5*^, MBBS, PhD; Peter H. Dixon^5^, PhD.

1. Institute for Women’s Health, Population Health Sciences, University College London, London, UK.

2. Institute for Population Health Sciences, Barts and the London School of Medicine and Dentistry, Queen Mary University of London, London, UK.

3. Centre for Cell Biology and Cutaneous Research, Blizard Institute, Barts and the London School of Medicine and Dentistry, Queen Mary University of London, London, UK.

4. Blizard Institute, Barts and the London School of Medicine and Dentistry, Queen Mary University of London, London, UK.

5. Department of Women and Children’s Health, School of Life Course Sciences, King’s College London, London, UK.

*Correspondence to:

Catherine Williamson, catherine.williamson@kcl.ac.uk, 2.30W Hodgkin Building, Department of Women and Children’s Health, School of Life Course Sciences, FOLSM, King’s College London, Guy’s Campus, London SE1 1UL, UK

**Supplementary tables**

**Supplementary table 1. *Summary of the number of volunteers that carried more than one variant between the 5 gene candidates.***

**Supplementary table 2. *Summary of all variants identified including clinical, literature review, protein function and in-silico variant prediction.***

**Supplementary table 3. *Variants identified in volunteers with raised TSBA concentrations but no diagnosis of ICP based on electronic health records.***

**Supplementary table 4. *ABCB4 variants identified with a cholestatic phenotype reported in the literature or no phenotype previously reported.***

**Supplementary table 5. *ABCB11 variants identified with a cholestatic phenotype reported in the literature or no phenotype previously reported.***

**Supplementary table 6. *ATP8B1 variants identified with a cholestatic phenotype reported in the literature or no phenotype previously reported.***

**Supplementary table 7. *NR1H4 variants identified with no cholestatic phenotype previously reported.***

**Supplementary table 8. *TJP2 variants identified with a cholestatic phenotype reported in the literature or no phenotype previously reported.***

**Supplementary table 9. *Summary of protein modelling results in variants that passed inclusion criteria and had a resolved 3D structure.***

**Supplementary table 1. *Summary of the number of volunteers that carried more than one variant between the 5 gene candidates.***

|  | **ABCB4** | **ABCB11** | **ATP8B1** | **NR1H4** | **TJP2** |
| --- | --- | --- | --- | --- | --- |
| **ABCB4** | **1** | **6** | **1** | **2** | **5** |
| **ABCB11** | **6** | **5** | **2** | **1** | **4** |
| **ATP8B1** | **1** | **2** | **0** | **0** | **3** |
| **NR1H4** | **2** | **1** | **0** | **1** | **0** |
| **TJP2** | **5** | **4** | **3** | **0** | **7** |

**Supplementary table 2. *Summary of all variants identified including clinical, literature review, protein function and in-silico variant prediction.***

|  | **SNV** | | | | | **Protein change** | | **Protein function prediction** | | | ***In-silico* variant prediction** | | | | | | | |
| --- | --- | --- | --- | --- | --- | --- | --- | --- | --- | --- | --- | --- | --- | --- | --- | --- | --- | --- |
|  | **CHR** | **POS** | **REF** | **ALT** |  | | **Conserved** | | **Disease propensity** | **Other variants in region** | **Varsome** | **SIFT** | **PolyPhen** | **CADD** | **Revel** | **MetaLR** | **Meta_SVM** | **M_CAP** |
| ABCB4 | chr7 | 87402176 | C | T | G1254S | | 1 | | 1 | 0 | 1 | deleterious(0) | probably damaging(1) | 26.3 | 0.94802 | D | D | D |
|  | chr7 | 87408168 | G | A | P1050S | | 1 | | 0 | 1 | 1 | tolerated(0.21) | possibly damaging(0.767) | 22.8 | 0.6331 | D | D | D |
|  | chr7 | 87417497 | C | T | A833T | | 0 | | 0 | 1 | 1 | deleterious(0) | probably damaging(1) | 27 | 0.88496 | D | D | D |
|  | chr7 | 87440230 | T | C | N510S | | 1 | | 0 | 1 | 1 | deleterious(0.02) | possibly damaging(0.854) | 23.1 | 0.82556 | D | D | D |
|  | chr7 | 87426810 | C | G | Q668H | | 0 | | 0 | 0 | 3 | tolerated(0.31) | benign(0) | 6.848 | 0.60481 | T | T | D |
|  | chr7 | 87406364 | C | T | R1137Q | | 0 | | 1 | 0 | 1 | deleterious(0.02) | possibly damaging(0.46) | 26.2 | 0.87877 | D | D | D |
|  | chr7 | 87420029 | C | T,A | R788L | | 0 | | 1 | 1 | 1 | deleterious(0) | Probably damaging(0.982) | 23.6 | 0.88684 | T | T | D |
|  | chr7 | 87426862 | G | T | T651N | | 0 | | 0 | 1 | 3 | tolerated(0.43) | benign(0.038) | 0.999 | 0.423 | T | T | D |
|  | chr7 | 87443722 | T | C | K391E | | 1 | | 0 | 1 | 3 | tolerated(0.23) | benign(0.35) | 19.78 | 0.66922 | D | T | D |
|  | chr7 | 87418539 | C | T | G826R | | 1 | | 1 | 0 | 1 | deleterious(0) | probably damaging(1) | 33 | 0.95499 | D | D | D |
|  | chr7 | 87426786 | C | T | M676I | | 0 | | 0 | 0 | 2 | tolerated(0.44) | benign(0) | 1.525 | 0.51995 | T | T | D |
|  | chr7 | 87426758 | C | T | D686N | | 0 | | 0 | 1 | 2 | tolerated(0.22) | benign(0) | 1.98 | 0.32697 | T | T | D |
|  | chr7 | 87454542 | T | C | M113V | | 1 | | 0 | 1 | 1 | deleterious(0.04) | benign(0.079) | 22.8 | 0.80713 | T | T | D |
|  | chr7 | 87420068 | G | A | T775M | | 1 | | 1 | 1 | 2 | tolerated(0.5) | probably damaging(0.944) | 22.7 | 0.8124 | D | T | D |
|  | chr7 | 87439833 | A | G | F522S | | 1 | | 1 | 1 | 1 | deleterious(0) | probably damaging(0.92) | 28.3 | 0.96627 | D | D | D |
|  | chr7 | 87440360 | A | C | Y467D | | 1 | | 1 | 1 | 1 | deleterious(0.01) | possibly damaging(0.674) | 23.8 | 0.83394 | D | D | D |
|  | chr7 | 87449993 | C | G | G270R | | 1 | | 1 | 0 | 1 | deleterious(0.01) | probably damaging(0.933) | 26.9 | 0.92964 | D | D | D |
|  | chr7 | 87426778 | T | C | K679R | | 0 | | 0 | 0 | 2 | tolerated(0.6) | benign(0) | 4.349 | 0.45696 | T | T | D |
|  | chr7 | 87406456 | C | G | Q1106H | | 2 | | 0 | 1 | 1 | tolerated(0.09) | benign(0.085) | 22.5 | 0.62767 | D | D | D |
|  | chr7 | 87431528 | C | T | R590Q | | 1 | | 1 | 1 | 4 | deleterious(0.01) | probably damaging(0.967) | 31 | 0.89829 | D | D | . |
|  | chr7 | 87453110 | C | A | G124C | | 1 | | 1 | 1 | 1 | deleterious(0) | probably damaging(0.998) | 29.4 | 0.98549 | D | D | D |
|  | chr7 | 87462827 | G | C | L73V | | 1 | | 0 | 1 | 1 | tolerated(0.3) | benign(0.29) | 14.12 | 0.60123 | D | T | D |
|  | chr7 | 87426841 | C | A | W658L | | 0 | | 1 | 1 | 2 | tolerated(0.65) | benign(0) | 14.87 | 0.5927 | T | T | D |
|  | chr7 | 87406437 | C | T | G1113R | | 1 | | 1 | 1 | 1 | deleterious(0) | probably damaging(1) | 31 | 0.97885 | D | D | D |
|  | chr7 | 87431565 | C | A | A578S | | 1 | | 0 | 1 | 1 | deleterious(0) | probably damaging(0.936) | 33 | 0.88963 | D | D | D |
|  | chr7 | 87406316 | A | T | I1153K | | 1 | | 1 | 0 | 1 | deleterious(0) | possibly damaging(0.887) | 27 | 0.99443 | D | D | D |
|  | chr7 | 87406404 | A | G | C1124R | | 1 | | 1 | 0 | 1 | tolerated(0.1) | probably damaging(0.99) | 25.9 | 0.93475 | D | D | D |
|  | chr7 | 87408039 | C | T | V1093M | | 0 | | 0 | 1 | 1 | tolerated(0.05) | probably damaging(0.962) | 26.1 | 0.9211 | D | D | D |
|  | chr7 | 87409306 | G | T | A1004E | | 0 | | 1 | 0 | 2 | deleterious(0) | probably damaging(0.971) | 28.4 | 0.96919 | D | D | D |
|  | chr7 | 87451757 | C | A,T | V192I | | 0 (0) | | 1 (0) | 1 (1) | 1 | tolerated(1) | benign(0.005) | 16.12 | 0.52596 | T | T | D |
|  | chr7 | 87402223 | T | C | N1238S | | 1 | | 0 | 0 | 2 | deleterious(0.04) | possibly damaging(0.559) | 15.09 | 0.60717 | T | T | D |
|  | chr7 | 87422179 | A | G | I753T | | 0 | | 0 | 1 | 2 | tolerated(0.23) | benign(0.003) | 19.75 | 0.59021 | T | T | D |
|  | chr7 | 87422195 | G | C | Q748E | | 0 | | 0 | 1 | 2 | tolerated(0.19) | benign(0.02) | 21.2 | 0.63418 | T | D | D |
|  | chr7 | 87472653 | T | C | K35E | | 0 | | 0 | 1 | 2 | tolerated(0.94) | benign(0.003) | 15.86 | 0.49989 | T | T | D |
|  | chr7 | 87408137 | T | C | K1060R | | 0 | | 0 | 1 | 2 | tolerated(0.12) | benign(0.062) | 22.7 | 0.48707 | D | T | D |
|  | chr7 | 87426763 | T | A | E684V | | 0 | | 1 | 0 | 2 | tolerated(0.06) | benign(0.007) | 16.25 | 0.53625 | D | T | D |
|  | chr7 | 87444956 | A | G | I342T | | 1 | | 0 | 1 | 3 | deleterious(0.02) | benign(0.061) | 23.4 | 0.74093 | T | T | D |
|  | chr7 | 87453040 | C | T | R147K | | 0 | | 0 | 1 | 2 | tolerated(1) | benign(0) | 9.241 | 0.5602 | T | T | D |
|  | chr7 | 87402265 | C | T | R1224H | | 1 | | 1 | 0 | 1 | deleterious(0.01) | probably damaging(0.994) | 31 | 0.9328 | D | D | D |
|  | chr7 | 87418605 | C | T | D804N | | 1 | | 0 | 0 | 1 | deleterious(0.01) | possibly damaging(0.767) | 32 | 0.92438 | D | D | D |
|  | chr7 | 87452957 | T | C | T175A | | 1 | | 0 | 1 | 3 | tolerated(0.05) | possibly damaging(0.615) | 24 | 0.83339 | T | D | . |
| ABCB11 | chr2 | 168923738 | C | T | D1284N | | 0 | | 0 | 1 | 1 | deleterious(0.03) | benign(0.057) | 23.5 | 0.76289 | T | T | D |
|  | chr2 | 168932441 | C | T | R1050H | | 0 | | 1 | 1 | 2 | tolerated(0.9) | probably damaging(0.912) | 23.2 | 0.79395 | D | D | D |
|  | chr2 | 168990858 | A | G | V284A | | 2 | | 0 | 1 | 1 | tolerated(0.15) | probably damaging(0.93) | 24.6 | 0.8887 | D | D | D |
|  | chr2 | 168970082 | T | C | N591S | | 1 | | 0 | 1 | 4 | tolerated(0.06) | possibly damaging(0.883) | 24 | 0.61416 | T | T | . |
|  | Hom (n) | 99 | Het (n) | 760 |  |  |  |  |  |  |  |  |  |  |  |  |  |  |
|  | chr2 | 168968473 | T | C | M677V | | 0 | | 0 | 1 | 4 | tolerated(0.46) | benign(0) | 0.716 | 0.50616 | T | T | . |
|  | Het (n) | 99 | Hom (n) | 3 |  |  |  |  |  |  |  |  |  |  |  |  |  |  |
|  | chr2 | 168923810 | C | G | A1260P | | 1 | | 1 | 1 | 1 | deleterious(0) | probably damaging(0.949) | 27.3 | 0.97698 | D | D | D |
|  | chr2 | 168935313 | T | C | Q976R | | 1 | | 0 | 1 | 2 | tolerated(0.58) | benign(0.001) | 0.622 | 0.42115 | T | T | D |
|  | chr2 | 168936268 | C | A | A926S | | 0 | | 0 | 1 | 1 | tolerated(0.06) | probably damaging(0.976) | 23.6 | 0.81356 | D | D | D |
|  | chr2 | 168968466 | G | A | A679V | | 0 | | 0 | 0 | 2 | tolerated(0.29) | benign(0.001) | 4.859 | 0.59146 | T | T | D |
|  | chr2 | 168971870 | T | C | N539D | | 1 | | 0 | 1 | 2 | tolerated(1) | benign(0.034) | 19.33 | 0.62325 | T | T | D |
|  | chr2 | 168972026 | G | A | R487C | | 1 | | 1 | 1 | 1 | deleterious(0) | probably damaging(0.991) | 33 | 0.93631 | D | D | D |
|  | chr2 | 168986262 | C | T | A311T | | 0 | | 0 | 0 | 1 | deleterious(0) | probably damaging(0.975) | 26 | 0.90885 | D | D | D |
|  | chr2 | 169013378 | C | T | V95I | | 0 | | 0 | 1 | 3 | tolerated(1) | benign(0.003) | 0.919 | 0.43033 | T | T | D |
|  | chr2 | 169013381 | C | T | D94N | | 0 | | 0 | 1 | 1 | tolerated(0.13) | possibly damaging(0.815) | 22.7 | 0.72016 | T | T | D |
|  | chr2 | 169018091 | T | C | K12R | | 0 | | 0 | 0 | 2 | tolerated_low_confidence(0.19) | benign(0.007) | 20.4 | 0.47225 | T | T | D |
|  | chr2 | 168923741 | C | A | A1283S | | 1 | | 0 | 1 | 2 | tolerated(0.3) | benign(0.005) | 18.14 | 0.70014 | D | D | D |
|  | chr2 | 168923785 | C | T | R1268Q | | 1 | | 1 | 0 | 1 | deleterious(0) | probably damaging(0.999) | 29.4 | 0.97997 | D | D | D |
|  | chr2 | 168924718 | C | T | R1235Q | | 1 | | 1 | 1 | 1 | deleterious(0.02) | probably damaging(1) | 28.9 | 0.86753 | D | D | D |
|  | chr2 | 168927259 | T | C | D1172G | | 1 | | 0 | 0 | 1 | deleterious(0.01) | probably damaging(0.957) | 29.7 | 0.99039 | D | D | D |
|  | chr2 | 168927316 | C | T | R1153H | | 2 | | 1 | 1 | 1 | deleterious(0.01) | probably damaging(1) | 27.5 | 0.97512 | D | D | D |
|  | chr2 | 168932442 | G | A | R1050C | | 1 | | 1 | 1 | 1 | deleterious(0.02) | probably damaging(0.939) | 32 | 0.97475 | D | D | D |
|  | chr2 | 168935256 | G | A | A995V | | 0 | | 0 | 1 | 1 | tolerated(0.1) | possibly damaging(0.833) | 23.7 | 0.78535 | D | D | D |
|  | chr2 | 168936261 | C | T | R928Q | | 1 | | 1 | 1 | 2 | tolerated(0.55) | benign(0.003) | 13.29 | 0.80058 | T | T | D |
|  | chr2 | 168964232 | T | C | K718E | | 0 | | 0 | 1 | 2 | tolerated(0.3) | benign(0) | 15.19 | 0.60123 | T | T | D |
|  | chr2 | 168969514 | C | T | R616H | | 1 | | 1 | 0 | 1 | deleterious(0) | probably damaging(0.999) | 24.9 | 0.91611 | D | D | D |
|  | chr2 | 168972022 | G | C | S488C | | 1 | | 0 | 0 | 1 | deleterious(0) | probably damaging(0.935) | 26 | 0.8124 | D | D | D |
|  | chr2 | 168973761 | G | A | T463I | | 0 | | 0 | 1 | 1 | deleterious(0) | probably damaging(0.991) | 28.3 | 0.98834 | D | D | D |
|  | chr2 | 168995438 | C | T | M174I | | 1 | | 0 | 0 | 2 | tolerated(1) | benign(0) | 8.761 | 0.5046 | T | T | D |
|  | chr2 | 169013314 | T | C | N116S | | 0 | | 0 | 1 | 1 | tolerated(0.07) | probably damaging(0.965) | 22.9 | 0.81761 | D | D | D |
|  | chr2 | 169013431 | G | A | P77L | | 0 | | 0 | 1 | 1 | deleterious(0) | probably damaging(0.976) | 27.5 | 0.95935 | D | D | D |
|  | chr2 | 168923626 | C | T | S1321N | | 1 | | 0 | 0 | 2 | deleterious_low_confidence(0) | probably damaging(0.998) | 24.7 | 0.81761 | D | D | D |
|  | chr2 | 168923728 | G | A | A1287V | | 1 | | 0 | 0 | 2 | tolerated(0.58) | benign(0.127) | 20.5 | 0.52296 | T | T | D |
|  | chr2 | 168923740 | G | A | A1283V | | 1 | | 0 | 1 | 1 | deleterious(0) | benign(0.311) | 24.7 | 0.84535 | D | D | D |
|  | chr2 | 168924788 | C | T | V1212I | | 1 | | 0 | 0 | 1 | deleterious(0.01) | probably damaging(0.999) | 26 | 0.80238 | D | D | D |
|  | chr2 | 168924791 | T | C | N1211D | | 1 | | 0 | 0 | 2 | tolerated(0.28) | benign(0.052) | 22.3 | 0.58135 | T | T | D |
|  | chr2 | 168927236 | T | C | T1180A | | 2 | | 0 | 0 | 2 | deleterious(0.01) | benign(0.338) | 23.3 | 0.64157 | T | T | D |
|  | chr2 | 168944621 | G | A | A865V | | 2 | | 0 | 1 | 4 | tolerated(0.07) | possibly damaging(0.647) | 26 | 0.81818 | D | D | . |
|  | chr2 | 168958110 | G | C | Q733E | | 1 | | 0 | 0 | 2 | tolerated(1) | benign(0) | 8.506 | 0.40989 | T | T | D |
|  | chr2 | 168964249 | A | G | L712S | | 0 | | 1 | 0 | 2 | tolerated(0.42) | benign(0.001) | 16.74 | 0.5602 | T | T | D |
|  | chr2 | 168964259 | C | T | E709K | | 0 | | 1 | 0 | 2 | tolerated(0.24) | benign(0.036) | 22.2 | 0.54201 | T | T | D |
|  | chr2 | 168969409 | A | G | F651S | | 0 | | 1 | 0 | 1 | tolerated(0.08) | probably damaging(0.999) | 25.8 | 0.83339 | T | T | D |
|  | chr2 | 168969515 | G | A | R616C | | 1 | | 1 | 1 | 1 | deleterious(0) | probably damaging(1) | 28.9 | 0.97327 | D | D | D |
|  | chr2 | 168979903 | C | T | R387H | | 0 | | 1 | 0 | 2 | deleterious(0) | possibly damaging(0.661) | 26.2 | 0.89694 | D | D | D |
|  | chr2 | 168986255 | C | A | R313L | | 0 | | 1 | 0 | 2 | deleterious(0.05) | benign(0.007) | 22.3 | 0.54769 | T | T | D |
|  | chr2 | 168993761 | T | A | I245F | | 0 | | 1 | 1 | 1 | deleterious(0) | possibly damaging(0.784) | 26.7 | 0.88823 | D | D | D |
|  | chr2 | 168995388 | T | G | D191A | | 0 | | 1 | 1 | 1 | deleterious(0) | probably damaging(1) | 28.3 | 0.98183 | D | D | D |
|  | chr2 | 169013441 | T | C | I74V | | 1 | | 0 | 0 | 2 | tolerated(0.35) | benign(0.001) | 2.838 | 0.55468 | T | T | D |
|  | chr2 | 169018081 | C | G | E15D | | 0 | | 0 | 0 | 2 | tolerated(0.08) | benign(0.318) | 13.66 | 0.64157 | T | T | D |
| ATP8B1 | chr18 | 57691876 | C | T | R384H | | 2 | | 1 | 1 | 2 | deleterious(0.03) | possibly damaging(0.714) | 24.4 | 0.7727 | D | D | D |
|  | chr18 | 57650416 | A | G | V1161A | | 0 | | 0 | . | 2 | deleterious(0.04) | benign(0.051) | 24.2 | 0.51233 | T | T | D |
|  | chr18 | 57652159 | G | A | T1092I | | 0 | | 0 | 0 | 2 | tolerated(0.08) | probably damaging(0.977) | 25.9 | 0.82103 | T | T | D |
|  | chr18 | 57731768 | C | A | D14Y | | 1 | | 1 | . | 2 | deleterious_low_confidence(0) | possibly damaging(0.641) | 24.9 | 0.35136 | T | T | T |
|  | chr18 | 57684128 | A | G | I513T | | 0 | | 0 | 0 | 2 | deleterious(0.02) | possibly damaging(0.6) | 24.3 | 0.66064 | T | T | D |
|  | chr18 | 57655198 | G | T | A976E | | 1 | | 1 | 0 | 2 | deleterious(0) | possibly damaging(0.753) | 28.2 | 0.83725 | T | T | D |
|  | chr18 | 57661279 | C | T | V868I | | 1 | | 0 | 1 | 2 | deleterious(0) | probably damaging(0.996) | 26.6 | 0.83394 | D | D | D |
|  | chr18 | 57661335 | C | T | R849Q | | 0 | | 1 | . | 2 | tolerated(0.4) | benign(0.003) | 2.507 | 0.39643 | T | T | D |
|  | chr18 | 57662599 | T | C | R768G | | 0 | | 0 | 1 | 2 | tolerated(0.08) | benign(0.325) | 23.3 | 0.54344 | T | T | T |
|  | chr18 | 57668449 | A | G | V730A | | 1 | | 0 | 1 | 2 | deleterious(0) | benign(0.243) | 24.6 | 0.93901 | D | D | D |
|  | chr18 | 57688379 | T | C | Y450C | | 1 | | 1 | 1 | 2 | deleterious(0) | probably damaging(0.998) | 31 | 0.93475 | D | D | D |
|  | chr18 | 57691936 | G | T | A364E | | 0 | | 1 | 0 | 2 | tolerated(0.91) | benign(0.021) | 19.68 | 0.38257 | T | T | T |
|  | chr18 | 57695188 | C | G | G308A | | 2 | | 0 | 1 | 2 | deleterious(0) | probably damaging(1) | 25 | 0.95572 | D | D | D |
|  | chr18 | 57695189 | C | T | G308S | | 2 | | 1 | 1 | 2 | deleterious(0) | probably damaging(1) | 26.4 | 0.9833 | D | D | D |
|  | chr18 | 57697640 | C | T | V226M | | 1 | | 0 | 1 | 2 | deleterious(0) | probably damaging(0.999) | 25.2 | 0.9579 | D | D | D |
|  | chr18 | 57697682 | T | C | I212V | | 1 | | 0 | 1 | 2 | tolerated(0.29) | benign(0.038) | 17.71 | 0.50616 | T | T | T |
|  | chr18 | 57701087 | A | G | M169T | | 1 | | 0 | 0 | 2 | tolerated(0.08) | benign(0.119) | 22.9 | 0.70994 | T | T | D |
|  | chr18 | 57704618 | C | G | M110I | | 1 | | 0 | 0 | 2 | tolerated(0.26) | benign(0) | 20.9 | 0.63842 | T | T | T |
|  | chr18 | 57706584 | C | A | C62F | | 0 | | 1 | . | 2 | tolerated(0.63) | benign(0.029) | 17.92 | 0.66639 | T | T | D |
|  | chr18 | 57652563 | T | A | Q1061L | | 0 | | 0 | 0 | 2 | tolerated(0.2) | benign(0.261) | 23.3 | 0.45868 | T | T | T |
|  | chr18 | 57655354 | T | C | Y924C | | 0 | | 1 | 1 | 2 | deleterious(0) | possibly damaging(0.603) | 25.6 | 0.94015 | D | D | D |
|  | chr18 | 57662511 | C | T | R797H | | 0 | | 1 | 1 | 2 | tolerated(0.52) | benign(0.017) | 22.4 | 0.41556 | T | T | T |
|  | chr18 | 57671517 | C | T | R628Q | | 1 | | 1 | 1 | 1 | deleterious(0.01) | probably damaging(0.998) | 31 | 0.87539 | D | D | D |
|  | chr18 | 57688442 | T | G | E429A | | 0 | | 0 | 0 | 3 | tolerated(0.42) | benign(0.003) | 22.4 | 0.36411 | T | T | T |
|  | chr18 | 57704566 | G | C | L128V | | 2 | | 0 | 1 | 2 | tolerated(0.06) | probably damaging(0.991) | 23.8 | 0.74235 | T | T | D |
|  | chr18 | 57731674 | T | G | N45T | | 0 | | 0 | . | 4 | tolerated_low_confidence(0.11) | benign(0.112) | 15.45 | 0.28106 | T | T | T |
|  | chr18 | 57669394 | A | G | M674T | | 0 | | 0 | 1 | 4 | tolerated(0.5) | benign(0) | 12.04 | 0.42115 | T | T | . |
|  | Het (n) | 62 | Hom (n) | 2 |  |  |  |  |  |  |  |  |  |  |  |  |  |  |
|  | chr18 | 57706535 | G | C | H78Q | | 0 | | 0 | . | 4 | tolerated(0.66) | benign(0) | 0.002 | 0.16033 | T | T | . |
|  | Het (n) | 47 | Hom (n) | 1 |  |  |  |  |  |  |  |  |  |  |  |  |  |  |
|  | chr18 | 57674924 | T | C | I577V | | 1 | | 0 | 1 | 4 | tolerated(0.11) | possibly damaging(0.787) | 20.3 | 0.57094 | T | T | . |
|  | chr18 | 57706561 | C | T | D70N | | 1 | | 0 | . | 2 | tolerated(0.23) | probably damaging(0.926) | 23.7 | 0.52745 | T | T | D |
|  | Het (n) | 24 | Hom (n) | 1 |  |  |  |  |  |  |  |  |  |  |  |  |  |  |
|  | chr18 | 57661383 | C | T | R833Q | | 0 | | 1 | 1 | 4 | tolerated(0.55) | benign(0) | 16.99 | 0.23497 | T | T | . |
| NR1H4 | chr12 | 100532530 | T | C | M173T | | 1 | | 0 | 1 | 4 | deleterious(0) | probably damaging(0.989) | 25.6 | 0.95133 | D | D | D |
|  | chr12 | 100540812 | A | C | N358H | | 2 | | 0 | 0 | 2 | tolerated(0.31) | benign(0.417) | 22.6 | 0.67661 | D | D | D |
|  | chr12 | 100510849 | A | G | N51D | | 0 | | 0 | . | 2 | deleterious(0.02) | benign(0.015) | 20.1 | 0.5491 | D | T | D |
|  | chr12 | 100536552 | A | G | H258R | | 0 | | 0 | 0 | 2 | tolerated(0.16) | benign(0) | 14.75 | 0.4655 | D | T | D |
|  | chr12 | 100540688 | C | G | D316E | | 0 | | 0 | 0 | 2 | deleterious(0.05) | probably damaging(0.989) | 23.2 | 0.86453 | D | D | D |
|  | chr12 | 100561926 | A | G | K374E | | 1 | | 0 | 0 | 2 | deleterious(0.01) | probably damaging(0.997) | 27.6 | 0.92804 | D | D | D |
|  | chr12 | 100510831 | C | T | P45S | | 1 | | 0 | . | 2 | tolerated(0.11) | benign(0) | 7.036 | 0.44648 | D | T | D |
|  | chr12 | 100532555 | G | T | E181D | | 2 | | 0 | 1 | 2 | tolerated(0.3) | benign(0.024) | 16.04 | 0.70014 | D | T | D |
|  | chr12 | 100534956 | A | T | Q222L | | 0 | | 0 | 0 | 2 | tolerated(0.67) | benign(0.015) | 19.92 | 0.66064 | D | T | D |
| TJP2 | chr9 | 69236102 | A | G | T377A | | 1 | | 0 | 0 | 2 | tolerated(0.44) | benign(0.167) | 19.82 | 0.12947 | T | T | T |
|  | chr9 | 69205232 | G | A | R21H | | 0 | | 1 | 0 | 4 | tolerated(0.55) | benign(0.003) | 1.869 | 0.06539 | T | T | . |
|  | Het (n) | 443 | Hom (n) | 16 |  |  |  |  |  |  |  |  |  |  |  |  |  |  |
|  | chr9 | 69220926 | C | A | Q105K | | 0 | | 0 | 1 | 4 | tolerated(1) | benign(0.087) | 6.897 | 0.19598 | T | T | . |
|  | chr9 | 69212579 | A | G | Q8R | | 1 | | 0 | 0 | 2 | deleterious(0) | probably damaging(0.992) | 26.2 | 0.83172 | T | T | D |
|  | chr9 | 69218289 | C | A | T68N | | 0 | | 0 | 0 | 2 | deleterious(0.02) | possibly damaging(0.765) | 24.2 | 0.48053 | T | T | T |
|  | chr9 | 69221068 | C | T | P152L | | 0 | | 0 | . | 3 | tolerated(0.5) | benign(0.007) | 13.16 | 0.0584 | T | T | T |
|  | chr9 | 69227812 | C | T | R178C | | 0 | | 1 | 0 | 2 | deleterious(0) | probably damaging(0.91) | 26.7 | 0.37035 | T | T | D |
|  | chr9 | 69229220 | G | A | R255H | | 0 | | 1 | . | 2 | tolerated(0.07) | benign(0.029) | 23.7 | 0.04481 | T | T | T |
|  | chr9 | 69237065 | G | C | R461P | | 1 | | 1 | 0 | 2 | deleterious(0) | probably damaging(1) | 31 | 0.75074 | T | T | D |
|  | chr9 | 69248118 | C | T | T902M | | 2 | | 1 | . | 2 | deleterious(0) | probably damaging(1) | 26.1 | 0.6757 | T | T | D |
|  | chr9 | 69251321 | G | A | R1070K | | 0 | | 0 | . | 2 | tolerated_low_confidence(0.11) | benign(0.019) | 27.6 | 0.5046 | T | T | D |
|  | chr9 | 69174379 | G | C | V3L | | 1 | | 0 | 0 | 2 | deleterious_low_confidence(0) | possibly damaging(0.477) | 25.2 | 0.32006 | T | T | D |
|  | chr9 | 69205273 | C | A | L38I | | . | | . | . | 2 | tolerated_low_confidence(0.16) | benign(0.034) | 0.507 | 0.00703 | T | T | T |
|  | chr9 | 69216363 | G | T | A24S | | 1 | | 0 | 0 | 2 | deleterious(0) | probably damaging(0.997) | 29.6 | 0.70427 | T | T | D |
|  | chr9 | 69234509 | G | A | G339D | | 1 | | 1 | 0 | 2 | deleterious(0) | probably damaging(0.976) | 29.1 | 0.88963 | T | D | D |
|  | chr9 | 69248193 | C | T | T927I | | 1 | | 0 | . | 2 | deleterious(0) | benign(0.022) | 24.8 | 0.22919 | T | T | T |
|  | chr9 | 69254222 | G | C | E1142Q | | 0 | | 0 | . | 2 | deleterious(0.02) | probably damaging(0.979) | 26.6 | 0.5519 | T | T | D |
|  | chr9 | 69254357 | C | T | R1187W | | 1 | | 1 | . | 2 | deleterious_low_confidence(0) | probably damaging(1) | 24.8 | 0.49511 | T | T | D |
|  | chr9 | 69216385 | A | G | N31S | | 1 | | 0 | 1 | 2 | deleterious(0.01) | probably damaging(0.985) | 25.6 | 0.59638 | T | T | D |
|  | chr9 | 69216409 | C | T | T39M | | 1 | | 1 | 1 | 2 | deleterious(0.04) | probably damaging(0.999) | 25 | 0.40608 | T | T | T |
|  | chr9 | 69216454 | G | C | G54A | | 2 | | 0 | 1 | 2 | deleterious(0) | probably damaging(0.999) | 27.1 | 0.74021 | T | T | D |
|  | chr9 | 69216456 | C | A | L55M | | 1 | | 0 | 0 | 2 | deleterious(0.04) | probably damaging(0.999) | 23.2 | 0.36201 | T | T | T |
|  | chr9 | 69218297 | G | A | E71K | | 0 | | 1 | 1 | 2 | deleterious(0) | possibly damaging(0.658) | 28.7 | 0.59393 | T | T | T |
|  | chr9 | 69218318 | G | T | A78S | | 1 | | 0 | 1 | 2 | deleterious(0.02) | possibly damaging(0.801) | 27.3 | 0.81298 | T | T | D |
|  | chr9 | 69218351 | G | A | A89T | | 0 | | 0 | 1 | 2 | deleterious(0) | possibly damaging(0.735) | 28.9 | 0.92601 | T | T | D |
|  | chr9 | 69221403 | C | A | R45S | | 0 | | 0 | . | 2 | deleterious(0.02) | benign(0.165) | 22.1 | 0.32697 | T | T | D |
|  | chr9 | 69225355 | C | T | T93M | | 2 | | 1 | 0 | 2 | deleterious(0.02) | benign(0.286) | 24.5 | 0.5976 | T | T | D |
|  | chr9 | 69227983 | C | T | S199F | | 1 | | 0 | 0 | 2 | deleterious(0.01) | possibly damaging(0.681) | 24.2 | 0.19911 | T | T | T |
|  | chr9 | 69228083 | C | T,G | N232K | | 0 | | 1 | . | 2 | tolerated(0.81) | benign(0) | 7.87 | 0.06539 | T | T | T |
|  | chr9 | 69236130 | G | T | G386V | | 1 | | 1 | 0 | 2 | deleterious(0) | probably damaging(1) | 28.4 | 0.88448 | T | T | D |
|  | chr9 | 69237073 | C | T | L464F | | 0 | | 0 | . | 2 | deleterious(0) | probably damaging(1) | 24.4 | 0.44999 | T | T | D |
|  | chr9 | 69237097 | A | C | T472P | | 1 | | 1 | 0 | 2 | tolerated(0.09) | probably damaging(0.999) | 26.5 | 0.77141 | T | T | D |
|  | chr9 | 69238758 | G | A | R533Q | | 1 | | 1 | 0 | 2 | deleterious(0) | probably damaging(0.999) | 32 | 0.80653 | T | T | D |
|  | chr9 | 69239944 | A | T | H546L | | 1 | | 0 | 0 | 2 | deleterious(0) | benign(0.135) | 27.3 | 0.92397 | T | T | D |
|  | chr9 | 69246785 | G | A | G865R | | 1 | | 1 | . | 2 | deleterious(0) | probably damaging(0.982) | 29.8 | 0.69678 | T | T | T |
|  | chr9 | 69248052 | A | G | Y880C | | 1 | | 1 | . | 2 | deleterious(0.03) | benign(0.106) | 27.2 | 0.62547 | T | T | D |
|  | chr9 | 69251333 | T | G | M1074R | | 1 | | 0 | . | 2 | deleterious_low_confidence(0) | possibly damaging(0.791) | 25.2 | 0.4655 | T | T | D |

(Varsome prediction: 0, pathogenic; 1, likely pathogenic; 2, variant of unknown significance; 3, likely benign; 4, benign. Conserved: 0, not conserved; 1, highly conserved. Disease propensity: 0, low disease propensity; 1, high disease propensity. Other variants in region: 0, no; 1, yes. Abbreviations: ALT, alternative; CHR, chromosome; D, deleterious; POS, position; REF, reference; SNV, Single Nucleotide Variant; T, tolerant.)

**Supplementary table 3. *Variants identified in volunteers with raised TSBA concentrations but no diagnosis of ICP based on electronic health records.***

| All variants in patients with raised TSBA but no diagnosis of ICP | | | | | |
| --- | --- | --- | --- | --- | --- |
| **Volunteer** | **Gene** | **Variants** | **Zygosity** | **Type** | **Highest BA (µmol/L)** |
| 2 | ATP8B1 | M674T | het | Non-synonymous | 13.4 |
| 2 | ATP8B1 | I577V | het | Non-synonymous |  |
| 2 | TJP2 | Q105K | hom | Non-synonymous |  |
| 10 | ABCB4 | T175A | het | Non-synonymous | 11 |
| * 11 | TJP2 | R105K | het | Non-synonymous | 24 |
| 14 | TJP2 | Q105K | het | Non-synonymous | 11.1 |
| # 16 | ABCB11 | M677V | het | Non-synonymous | 14 |
| 17 | ABCB11 | V284A | het | Non-synonymous | 11 |
| * 24 | ABCB11 | N591S | het | Non-synonymous | 343 |
| 24 | ABCB4 | M113V | het | Non-synonymous |  |
| 24 | TJP2 | Q105K | hom | Non-synonymous |  |
| 27 | ABCB11 | N591S | het | Non-synonymous | 10 |
| 28 | NH1R4 | H258R | het | Non-synonymous | 13 |
| 36 | ABCB11 | M677V | hom | Non-synonymous | 11.2 |

(Abbreviations: Het, heterozygous; hom, homozygous; ICP, intrahepatic cholestasis of pregnancy; TSBA, total serum bile acids. Symbols: #, non-pregnant- history of gallstone, hepatitis C and cirrhosis of liver; *possible diagnosis of ICP. Please note, all other volunteers were identified to be pregnant but to not have a diagnosis of ICP.)

**Supplementary table 4. *ABCB4 variants identified with a cholestatic phenotype reported in the literature or no phenotype previously reported.***

| **Clinical phenotype** | **Gene** | **Transcript** | **Protein change** | **dbSNP** | **gnomAD AF** | **G&H AF*** | **ACMG-AMP classification** | **ACMG-AMP criteria** | **Clinvar** | **Ref** |
| --- | --- | --- | --- | --- | --- | --- | --- | --- | --- | --- |
| Cholestatic phenotype reported in the literature | ABCB4 | ENSP00000496956.1:p.Gln1106His | Q1106H ^ | rs779653372 | 0.00001219 | 0.00009889 | LP | PM1, PM2, PP2 | . | [1] |
|  | ABCB4 | ENSP00000496956.1:p.Thr775Met | T775M | rs14805219 | 0.00050410 | 0.00028774 | VUS | PM1, PM2, PP2, BP4 | . | [2-9] |
|  | ABCB4 | ENSP00000496956.1:p.Lys679Arg | K679R | rs374432576 | 0.00000813 | 0.00009568 | VUS | PM2, PP2, BP4 | . | [10] |
|  | ABCB4 | ENSP00000496956.1:p.Arg590Gln | R590Q | rs45575636 | 0.00449100 | 0.00029002 | Benign | PM1, PP2, PP3 | conflicting | [3, 4, 11-17] |
|  | ABCB4 | ENSP00000496956.1:p.Phe522Ser | F522S | . | . | 0.00009575 | LP | PM1, PM2, PP2, PP3 | . | [18] |
|  | ABCB4 | ENSP00000496956.1:p.Tyr467Asp | Y467D | rs1472679087 | 0.00000813 | 0.00009557 | LP | PM1, PM2. PP2 | . | [15] |
|  | ABCB4 | ENSP00000496956.1:p.Gly270Arg | G270R | rs551234479 | 0.00023170 | 0.00057296 | LP | PM1, PM2, PP2, PP3 | VUS | [19] |
|  | ABCB4 | ENSP00000496956.1:p.Met113Ile | M113V | rs752700100 | 0.00005719 | 0.00078705 | LP | PM1, PM2, PP2 | . | [1, 3] |
|  | ABCB4 | ENSP00000496956.1:p.Leu73Val | L73V | rs8187788 | 0.000707 | 0.00009569 | LP | PP2, PP5, BP4 | VUS | [7, 11, 12, 20-22] |
| No phenotype reported in the literature | ABCB4 | ENSP00000496956.1:p.Asn1238Ser | N1238S | . | . | 0.00010739 | VUS | PM1, PM2, PP2, BP4 | . | . |
|  | ABCB4 | ENSP00000496956.1:p.Arg1224His | R1224H | rs144790968 | 0.00003254 | 0.00009804 | LP | PM1, PM2, PP2, PP3 | . | . |
|  | ABCB4 | ENSP00000496956.1:p.Ile1153Lys | I1153K | . | . | 0.00009623 | LP | PM1, PM2, PP2, PP3 | . | . |
|  | ABCB4 | ENSP00000496956.1:p.Cys1124Arg | C1124R | . | . | 0.00009582 | LP | PM1, PM2, PP2, PP3 | . | . |
|  | ABCB4 | ENSP00000496956.1:p.Gly1113Arg | G1113R | rs1282338773 | . | 0.00019562 | LP | PM1, PM2, PP2, PP3 | VUS | . |
|  | ABCB4 | ENSP00000496956.1:p.Val1093Met | V1093M | . | . | 0.00018657 | LP | PM1, PM2, PP2, PP3 | . | . |
|  | ABCB4 | ENSP00000496956.1:p.Lys1060Arg | K1060R | . | . | 0.00028796 | VUS | PM1, PM2, PP2, BP4 | . | . |
|  | ABCB4 | ENSP00000496956.1:p.Ala1004Glu | A1004E | . | . | 0.00009564 | VUS | PM2, PP2, PP3 | . | . |
|  | ABCB4 | ENSP00000496956.1:p.Asp804Asn | D804N | rs762755781 | 0.00000406 | 0.00009590 | LP | PM1, PM2, PP2, PP3 | . | . |
|  | ABCB4 | ENSP00000496956.1:p.Ile753Thr | I753T | . | . | 0.00028719 | VUS | PM1, PM2, PP2, BP4 | . | . |
|  | ABCB4 | ENSP00000496956.1:p.Gln748Glu | Q748E | . | . | 0.00009584 | VUS | PM1, PM2, PP2, BP4 | . | . |
|  | ABCB4 | ENSP00000496956.1:p.Glu684Val | E684V | . | . | 0.00009569 | VUS | PM2, PP2, BP4 | . | . |
|  | ABCB4 | ENSP00000496956.1:p.Trp658Leu | W658L | . | . | 0.00009580 | VUS | PM2, PP2, BP4 | . | . |
|  | ABCB4 | ENSP00000496956.1:p.Ala578Ser | A578S | . | . | 0.00066079 | LP | PM1, PM2, PP2 | . | . |
|  | ABCB4 | ENSP00000496956.1:p.Ile342Thr | I342T | . | . | 0.00009758 | LB | PM1, PM2, PP2 | . | . |
|  | ABCB4 | ENSP00000496956.1:p.Val192Ile | V192I | . | . | 0.00009557 | LP | PM1, PM2, PP2, PP3 | . | . |
|  | ABCB4 | ENSP00000496956.1:p.Arg147Lys | R147K | . | . | 0.00009566 | VUS | PM1, PM2, PP2, BP4 | . | . |
|  | ABCB4 | ENSP00000496956.1:p.Gly124Cys | G124C | . | . | 0.00009569 | LP | PM1, PM2, PM5, PP2, PP3 | . | . |
|  | ABCB4 | ENSP00000496956.1:p.Lys35Glu | K35E | rs879209136 | . | 0.00018748 | VUS | PM2, PP2, BP4 | . | . |

(Variants were filtered and annotated if they met the following inclusion criteria (MAF < 5%): 1. associated with a phenotype; 2. known in the literature; 3. no recorded GnomAD allele frequency; 4. predicted to be likely pathogenic (LP) based on all 7 *in-silico* predictors. Abbreviations: AF, allele frequency; ACMG-AMP, American College of Medical Genetics and Genomics and the Association for Molecular Pathology (BP, benign supporting; PM, pathogenic moderate; PP, pathogenic supporting); G&H, Genes & Health; LP, likely pathogenic; Ref, references; VUS, variant of unknown significance. Symbols: *Allele frequency specific to East London Genes & Health cohort; ^ see Supplementary Figure 4 for an illustrative example.)

**Supplementary table 5. *ABCB11 variants identified with a cholestatic phenotype reported in the literature or no phenotype previously reported.***

| **Clinical Phenotype** | **Gene** | **Transcript** | **Protein change** | **dbSNP** | **gnomAD AF** | **G&H AF*** | **ACMG-AMP classification** | **ACMG-AMP criteria** | **Clinvar** | **Ref** |
| --- | --- | --- | --- | --- | --- | --- | --- | --- | --- | --- |
| Cholestatic phenotype reported in the literature | ABCB11 | ENSP00000497931.1:p.Arg1268Gln | R1268Q | rs72549394 | . | 0.00021983 | LP | PM1, PM2, PP2, PP3, PP5 | . | [23-26] |
|  | ABCB11 | ENSP00000497931.1:p.Arg1153His | R1153H | rs748862206 | 0.00000813 | 0.00009584 | LP | PM1, PM2, PM5, PP2, PP3, PP5 | pathogenic | [25-30] |
|  | ABCB11 | ENSP00000497931.1:p.Arg1050Cys | R1050C | rs72549398 | 0.00001259 | 0.00009562 | LP | PM2, PP2, PP3, PP5 | pathogenic | [23, 27, 31-36] |
|  | ABCB11 | ENSP00000497931.1:p.Ala995Val | A995V | rs868669576 | 0.00000406 | 0.00009551 | LP | PM1, PM2, PP2, PP3 | . | [15] |
|  | ABCB11 | ENSP00000497931.1:p.Arg928Gln | R928Q | rs200488448 | 0.00003257 | 0.00010113 | VUS | PM1, PM2, PP2, BP4 | VUS | [37] |
|  | ABCB11 | ENSP00000497931.1:p.Arg616His | R616H | rs777021400 | 0.00002045 | 0.00009787 | LP | PM1, PM2, PP2, PP3 | VUS | [38] |
|  | ABCB11 | ENSP00000497931.1:p.Thr463Ile | T463I | rs1163486377 | . | 0.00009628 | LP | PM1, PM2, PP2, PP3 | . | [25, 26] |
|  | ABCB11 | ENSP00000497931.1:p.Ser1321Asn | S1321N | rs201693189 | 0.00014620 | 0.00097447 | VUS | PM2, PP2, PP3 | . | [39] |
|  | ABCB11 | ENSP00000497931.1:p.Ala1283Val | A1283V | rs372886308 | 0.00006548 | 0.00009619 | LP | PM1, PM2, PP2, PP3 | VUS | [39] |
|  | ABCB11 | ENSP00000497931.1:p.Asn1211Asp | N1211D | rs762729346 | 0.00006544 | 0.00019110 | VUS | PM1, PM2, PP2 | . | [26] |
|  | ABCB11 | ENSP00000497931.1:p.Ala865Val | A865V | rs118109635 | 0.00108500 | 0.00057361 | Benign | PM1, PP2, PP3, BP6, BS1, BS2 | benign/likely benign | [17, 37, 39-46] |
|  | ABCB11 | ENSP00000497931.1:p.Leu712Ser | L712S | rs372939910 | 0.00002690 | 0.00028852 | VUS | PM1, PP2, BP4 | benign/likely benign | [47] |
|  | ABCB11 | ENSP00000497931.1:p.Glu709Lys | E709K | rs201800225 | 0.00021450 | 0.00019260 | VUS | PM2, PP2, BP4 | VUS | [10, 25] |
|  | ABCB11 | ENSP00000497931.1:p.Arg387His | R387H | rs372784355 | 0.00000813 | 0.00009626 | VUS | PM2, PP2, PP3 | . | [48] |
|  | | | | | | | | | | |
| No  phenotype reported in the literature | ABCB11 | ENSP00000497931.1:p.Ala1283Ser | A1283S | . | . | 0.00009636 | VUS | PM1, PM2, PP2, BP4 | . | . |
|  | ABCB11 | ENSP00000497931.1:p.Arg1235Gln | R1235Q | rs750033238 | 0.00001218 | 0.00019099 | LP | PM1, PM2, PP2, PP3 | . | . |
|  | ABCB11 | ENSP00000497931.1:p.Asp1172Gly | D1172G | . | . | 0.00009582 | LP | PM1, PM2, PP2, PP3 | . | . |
|  | ABCB11 | ENSP00000497931.1:p.Lys718Glu | K718E | . | . | 0.00009666 | VUS | PM2, PP2, BP4 | . | . |
|  | ABCB11 | ENSP00000497931.1:p.Ser488Cys | S488C | rs777671329 | 0.00000409 | 0.00009549 | LP | PM1, PM2, PP2, PP3 | . | . |
|  | ABCB11 | ENSP00000497931.1:p.Met174Ile | M174I | . | . | 0.00009617 | VUS | PM1, PM2, PP2, BP4 | . | . |
|  | ABCB11 | ENSP00000497931.1:p.Asn116Ser | N116S | . | . | 0.00009569 | LP | PM1, PM2, PP2 | . | . |
|  | ABCB11 | ENSP00000497931.1:p.Pro77Leu | P77L | . | . | 0.00019110 | LP | PM1, PM2, PP2, PP3 | . | . |
|  | ABCB11 | ENSP00000497931.1:p.Ala1287Val | A1287V | . | . | 0.00009601 | VUS | PM1, PM2, PP2 | . | . |
|  | ABCB11 | ENSP00000497931.1:p.Val1212Ile | V1212I | rs546906441 | 0.00003697 | 0.00028664 | LP | PM1, PM2, PP2, PP3 | VUS | . |
|  | ABCB11 | ENSP00000497931.1:p.Thr1180Ala | T1180A | . | . | 0.00009621 | VUS | PM1, PM2, PP2, BP4 | . | . |
|  | ABCB11 | ENSP00000497931.1:p.Gln733Glu | Q733E | . | . | 0.00013839 | VUS | PM2, PP2, BP4 | . | . |
|  | ABCB11 | ENSP00000497931.1:p.Phe651Ser | F651S | rs868080119 | . | 0.00009560 | LP | PM1, PM2, PP2, PP3 | . | . |
|  | ABCB11 | ENSP00000497931.1:p.Arg616Cys | R616C | rs369187042 | 0.00001227 | 0.00009745 | LP | PM1, PM2, PP2, PP3 | VUS | . |
|  | ABCB11 | ENSP00000497931.1:p.Arg313Leu | R313L | . | . | 0.00023585 | VUS | PM1, PM2, PP2, BP4 | . | . |
|  | ABCB11 | ENSP00000497931.1:p.Ile245Phe | I245F | . | . | 0.00009555 | LP | PM1, PM2, PP2, PP3 | . | . |
|  | ABCB11 | ENSP00000497931.1:p.Asp191Ala | D191A | . | . | 0.00009577 | LP | PM1, PM2, PP2, PP3 | . | . |
|  | ABCB11 | ENSP00000497931.1:p.Ile74Val | I74V | . | . | 0.00009555 | VUS | PM1, PM2, PP2, BP4 | . | . |
|  | ABCB11 | ENSP00000497931.1:p.Glu15Asp | E15D | . | . | 0.00009835 | VUS | PM2, PP2, BP4 | . | . |

(Variants were filtered and annotated if they met the following inclusion criteria (MAF < 5%): 1. associated with a phenotype; 2. known in the literature; 3. no recorded GnomAD allele frequency; 4. predicted to be likely pathogenic (LP) based on all 7 *in-silico* predictors. Abbreviations: AF, allele frequency; ACMG-AMP, American College of Medical Genetics and Genomics and the Association for Molecular Pathology (BP, benign supporting; PM, pathogenic moderate; PP, pathogenic supporting); G&H, Genes & Health; LP, likely pathogenic; Ref, references; VUS, variant of unknown significance. Symbols: *Allele frequency specific to East London Genes & Health cohort.)

**Supplementary table 6. *ATP8B1 variants identified with a cholestatic phenotype reported in the literature or no phenotype previously reported.***

| **Clinical phenotype** | **Gene** | **Transcript** | **Protein change** | **dbSNP** | **gnomAD AF*** | **G&H AF*** | **ACMG-AMP classification** | **ACMG-AMP criteria** | **Clinvar** | **Ref** |
| --- | --- | --- | --- | --- | --- | --- | --- | --- | --- | --- |
| Cholestatic phenotype reported in the literature | ATP8B1 | ENSP00000497896.1:p.Ala976Glu | A976E | . | . | 0.00009564 | VUS | PM2, PP2, PP3 | . | [49] |
|  | ATP8B1 | ENSP00000497896.1:p.Arg849Gln | R849Q | rs144656719 | 0.00009342 | 0.00057372 | VUS | PM2, PP2, BP4 | VUS | [50] |
|  | ATP8B1 | ENSP00000497896.1:p.Gly308Ser | G308S | rs1007521320 | 0.00000406 | 0.00009617 | LP | PM2, PM5, PP2, PP3 | . | [51-53] |
|  | ATP8B1 | ENSP00000497896.1:p.Tyr924Cys | Y924C | rs145287364 | 0.00003249 | 0.00009549 | VUS | PM2, PP2, PP3, BP6 | VUS | [49] |
|  | ATP8B1 | ENSP00000497896.1:p.Arg833Gln | R833Q ^1.^ | rs568134011 | 0.00135300 | 0.01042260 | Benign | PM2, PP2, BP4 | Benign | [49] |
|  | ATP8B1 | ENSP00000497896.1:p.Glu429Ala | E429A | rs34018205 | 0.00061740 | 0.00009858 | LB | PM2, PP2, BP4, BP6 | Conflicting | [48, 49, 54, 55] |
|  | ATP8B1 | ENSP00000497896.1:p.Asn45Thr | N45T | rs146599962 | 0.00471500 | 0.00047856 | Benign | PM1, PP2, PP5, BS1, BS2, BP4, BP6 | Likely benign | [3, 7, 39, 49, 56-60] |
| No phenotype reported in the literature | ATP8B1 | ENSP00000497896.1:p.Val868Ile | V868I | rs750735500 | 0.00002031 | 0.00009573 | VUS | PM2, PP2, PP3 | . | . |
|  | ATP8B1 | ENSP00000497896.1:p.Arg768Gly | R768G | . | . | 0.00009810 | VUS | PM2, PP2, BP4 | . | . |
|  | ATP8B1 | ENSP00000497896.1:p.Val730Ala | V730A | rs1302793202 | 0.00000406 | 0.00019146 | VUS | PM2, PP2, PP3 | . | . |
|  | ATP8B1 | ENSP00000497896.1:p.Tyr450Cys | Y450C | . | . | 0.00009741 | VUS | PM2, PP2, PP3 | . | . |
|  | ATP8B1 | ENSP00000497896.1:p.Ala364Glu | A364E | . | . | 0.00009557 | VUS | PM2, PP2, BP4 | . | . |
|  | ATP8B1 | ENSP00000497896.1:p.Gly308Ala | G308A | . | . | 0.00009628 | LP | PM2, PM5, PP2, PP3 | . | . |
|  | ATP8B1 | ENSP00000497896.1:p.Val226Met | V226M | rs1449849281 | 0.00000406 | 0.00010030 | VUS | PM2, PP2, PP3 | . | . |
|  | ATP8B1 | ENSP00000497896.1:p.Ile212Val | I212V | . | . | 0.00028907 | VUS | PM2, PP2, BP4 | . | . |
|  | ATP8B1 | ENSP00000497896.1:p.Met169Thr | M169T | . | . | 0.00009582 | VUS | PM2, PP2 | . | . |
|  | ATP8B1 | ENSP00000497896.1:p.Met110Ile | M110I | . | . | 0.00013214 | VUS | PM1, PM2, PP2, BP4 | . | . |
|  | ATP8B1 | ENSP00000497896.1:p.Cys62Phe | C62F | . | . | 0.00068752 | VUS | PM2, PP2, BP4 | . | . |
|  | ATP8B1 | ENSP00000497896.1:p.Gln1061Leu | Q1061L | . | . | 0.00009549 | VUS | PM2, PP2 | . | . |
|  | ATP8B1 | ENSP00000497896.1:p.Arg797His | R797H | rs776245959 | 0.00001219 | 0.00009566 | VUS | PM2, PP2, BP4 | . | . |
|  | ATP8B1 | ENSP00000497896.1:p.Arg628Gln | R628Q | rs747906077 | 0.00001218 | 0.00009558 | LP | PM2, PM5, PP2, PP3 | . | . |
|  | ATP8B1 | ENSP00000497896.1:p.Leu128Val | L128V | . | . | 0.00012324 | VUS | PM1, PM2, PP2 | . | . |

(Variants were filtered and annotated if they met the following inclusion criteria (MAF < 5%): 1. associated with a phenotype; 2. known in the literature; 3. no recorded GnomAD allele frequency; 4. predicted to be likely pathogenic (LP) based on all 7 *in-silico* predictors. Abbreviations: AF, allele frequency; ACMG-AMP, American College of Medical Genetics and Genomics and the Association for Molecular Pathology (BP, benign supporting; PM, pathogenic moderate; PP, pathogenic supporting); G&H, Genes & Health; LP, likely pathogenic; Ref, references; VUS, variant of unknown significance. Symbols: *Allele frequency specific to East London Genes & Health cohort; ^1.^ R833Q, Hom (n) 1, Het (n) 110.)

**Supplementary table 7. *NR1H4 variants identified with no cholestatic phenotype previously reported.***

| **Clinical phenotype** | **Gene** | **Transcript** | **Protein change** | **dbSNP** | **gnomAD AF*** | **G&H AF*** | **ACMG-AMP classification** | **ACMG-AMP criteria** | **Clinvar** | **Ref** |
| --- | --- | --- | --- | --- | --- | --- | --- | --- | --- | --- |
| No phenotype reported in the literature | NR1H4 | ENSP00000496908.1:p.Asn51Asp | N51D | . | . | 0.00009549 | VUS | PM2, BP4 | . | . |
|  | NR1H4 | ENSP00000496908.1:p.His258Arg | H258R | rs780917996 | . | 0.00038373 | VUS | PM2, BP4 | . | . |
|  | NR1H4 | ENSP00000496908.1:p.Asp316Glu | D316E | rs778153216 | 0.00010970 | 0.00038270 | VUS | PM2, PP3 | . | . |
|  | NR1H4 | ENSP00000496908.1:p.Lys374Glu | K374E | rs906730263 | . | 0.00017100 | VUS | PM2, PP3 | . | . |
|  | NR1H4 | ENSP00000496908.1:p.Pro45Ser | P45S | . | . | 0.00009553 | VUS | PM2, BP4 | . | . |
|  | NR1H4 | ENSP00000496908.1:p.Glu181Asp | E181D | . | . | 0.00009553 | VUS | PM1, PM2, PP3 | . | . |
|  | NR1H4 | ENSP00000496908.1:p.Gln222Leu | Q222L | . | . | 0.00009551 | VUS | PM2 | . | . |

(Variants were filtered and annotated if they met the following inclusion criteria (MAF < 5%): 1. associated with a phenotype; 2. known in the literature; 3. no recorded GnomAD allele frequency; 4. predicted to be likely pathogenic (LP) based on all 7 *in-silico* predictors. Ref, references. ACMG-AMP, American College of Medical Genetics and Genomics and the Association for Molecular Pathology. Abbreviations: AF, allele frequency; ACMG-AMP, American College of Medical Genetics and Genomics and the Association for Molecular Pathology (BP, benign supporting; PM, pathogenic moderate; PP, pathogenic supporting); G&H, Genes & Health; LP, likely pathogenic; Ref, references; VUS, variant of unknown significance. Symbols: *Allele frequency specific to East London Genes & Health cohort.)

**Supplementary table 8. *TJP2 variants identified with a cholestatic phenotype reported in the literature or no phenotype previously reported.***

| **Clinical phenotype** | **Gene** | **Transcript** | **Protein change** | **dbSNP** | **gnomAD AF** | **G&H AF*** | **ACMG-AMP classification** | **ACMG-AMP criteria** | **Clinvar** | **Ref** |
| --- | --- | --- | --- | --- | --- | --- | --- | --- | --- | --- |
| Cholestatic phenotype reported in the literature | TJP2 | ENSP00000497539.1:p.Val3Leu | V3L | rs758381001 | 0.00001343 | 0.00009966 | VUS | PM2, BP4 | . | [7] |
|  | TJP2 | ENSP00000496791.1:p.Thr39Met | T39M | rs138241615 | 0.00147400 | 0.00019309 | VUS | PM2, BP4 | . | [3, 61] |
|  | TJP2 | ENSP00000496791.1:p.Ala89Thr | A89T | rs144396411 | 0.00023970 | 0.00019194 | VUS | PM1, PP3 | Conflicting | [62] |
|  | TJP2 | ENSP00000497787.1:p.Thr93Met | T93M | rs548602675 | 0.00000813 | 0.00009579 | VUS | PM2, PP3 | . | [7] |
| No phenotype reported in the literature | TJP2 | ENSP00000496791.1:p.Gly865Arg | G865R | . | . | 0.00009558 | VUS | PM2, PP3 | . | . |
|  | TJP2 | ENSP00000438262.1:p.Leu38Ile | L38I | . | . | 0.00009643 | VUS | PM2, BP4 | . | . |
|  | TJP2 | ENSP00000496791.1:p.Ala24Ser | A24S | . | . | 0.00009568 | VUS | PM1, PM2, PP3 | . | . |
|  | TJP2 | ENSP00000497787.1:p.Gly339Asp | G339D | . | . | 0.00011364 | VUS | PM1, PM2, PP3 | . | . |
|  | TJP2 | ENSP00000496791.1:p.Thr927Ile | T927I | . | . | 0.00009677 | VUS | PM2, BP4 | . | . |
|  | TJP2 | ENSP00000497861.1:p.Glu1142Gln | E1142Q | . | . | 0.00009558 | VUS | PM1, PM2, PP3 | . | . |
|  | TJP2 | ENSP00000497861.1:p.Arg1187Trp | R1187W | rs112109886 | . | 0.00009968 | VUS | PM1, PM2, PP3 | . | . |
|  | TJP2 | ENSP00000496791.1:p.Asn31Ser | N31S | . | . | 0.00009579 | VUS | PM1, PM2, PP3 | . | . |
|  | TJP2 | ENSP00000496791.1:p.Gly54Ala | G54A | . | . | 0.00011756 | VUS | PM1, PM2, PP3 | . | . |
|  | TJP2 | ENSP00000496791.1:p.Leu55Met | L55M | . | . | 0.00023502 | VUS | PM1, PM2, BP4 | . | . |
|  | TJP2 | ENSP00000496791.1:p.Glu71Lys | E71K | . | . | 0.00009582 | VUS | PM1, PM2 | . | . |
|  | TJP2 | ENSP00000496791.1:p.Ala78Ser | A78S | . | . | 0.00019205 | VUS | PM1, PM2, PP3 | . | . |
|  | TJP2 | ENSP00000497787.1:p.Arg45Ser | R45S | . | . | 0.00009584 | VUS | PM2, BP4 | . | . |
|  | TJP2 | ENSP00000497787.1:p.Ser199Phe | S199F | . | . | 0.00009766 | VUS | PM2 | . | . |
|  | TJP2 | ENSP00000497787.1:p.Asn232Lys | N232K | . | . | 0.00019164 | VUS | PM2, BP4 | . | . |
|  | TJP2 | ENSP00000497787.1:p.Gly386Val | G386V | . | . | 0.00009562 | VUS | PM2, PP3 | . | . |
|  | TJP2 | ENSP00000497787.1:p.Leu464Phe | L464F | . | . | 0.00009817 | VUS | PM2, PP3 | . | . |
|  | TJP2 | ENSP00000497787.1:p.Thr472Pro | T472P | . | . | 0.00010119 | VUS | PM2, PP3 | . | . |
|  | TJP2 | ENSP00000497787.1:p.Arg533Gln | R533Q | . | . | 0.00009562 | VUS | PM2, PP3 | . | . |
|  | TJP2 | ENSP00000497787.1:p.His546Tyr | H546L | rs760920622 | . | 0.00038197 | VUS | PM2, PP3 | . | . |
|  | TJP2 | ENSP00000496791.1:p.Tyr880Cys | Y880C | . | . | 0.00019593 | VUS | PM2, PP3 | . | . |
|  | TJP2 | ENSP00000496791.1:p.Met1074Arg | M1074R | . | . | 0.00009566 | VUS | PM2 | . | . |

(Variants were filtered and annotated if they met the following inclusion criteria (MAF < 5%): 1. associated with a phenotype; 2. known in the literature; 3. no recorded GnomAD allele frequency; 4. predicted to be likely pathogenic (LP) based on all 7 *in-silico* predictors. Abbreviations: AF, allele frequency; ACMG-AMP, American College of Medical Genetics and Genomics and the Association for Molecular Pathology (BP, benign supporting; PM, pathogenic moderate; PP, pathogenic supporting); G&H, Genes & Health; LP, likely pathogenic; Ref, references; VUS, variant of unknown significance. Symbols: *Allele frequency specific to East London Genes & Health cohort.)

**Supplementary table 9. *Summary of protein modelling results in variants that passed inclusion criteria and had a resolved 3D structure.***

| **Gene** | **Clinical phenotype** | **Variant** | **Features of substitution site** | **CUPSAT** | **∆∆G DUET** | **∆∆G SDM** | **∆∆G mCSM** | **Dynamut** | **Depth (Å)** | **SNPMuSiC** |
| --- | --- | --- | --- | --- | --- | --- | --- | --- | --- | --- |
| ABCB4 | ICP | G1254S | Unresolved 3D structure region |  |  |  |  |  |  |  |
| ABCB4 | ICP | P1050S | Nucleotide binding domain 2 | destabilising, unfavourable torsion angle of Ser residue, ∆∆G -1.53kcal/mol | destabilising -0.066kcal/mol | stabilising, 0.760kcal/mol | destabilising, -0.691 kcal/mol | decrease in molecule flexibility ΔΔSVib ENCoM:  -0.164kcal/mol-1/K-1 | WT (3.5Å) to Mutant (4.0Å) | deleterious effect (0.6) thermodynamic stability - destabilising thermal stability - destabilising |
| ABCB4 | ICP | A833T | Transmembrane domain 2 | destabilising, unfavourable torsion angle of Thr residue, ∆∆G -0.83kcal/mol | destabilising -1.661kcal/mol | destabilising, -2.620kcal/mol | destabilising, -1.487 kcal/mol | decrease in molecule flexibility ΔΔSVib ENCoM:  -0.191kcal/mol-1/K-1 | WT (5.5Å) to Mutant (6.2Å) | neutral effect (-0.45) |
| ABCB4 | ICP | N510S | Nucleotide binding domain 1 | destabilising, unfavourable torsion angle of Ser residue, ∆∆G -0.63kcal/mol | destabilising -0.816kcal/mol | destabilising, -1.300kcal/mol | destabilising -0.688kcal/mol | increase in molecule flexibility  ΔΔSVib ENCoM: 0.361kcal/mol-1/K-1 | WT (3.6Å) to Mutant (3.3Å) | deleterious effect (0.1) thermodynamic stability - destabilising thermal stability - destabilising |
| ABCB4 | Gallstone disease | R1137Q | Nucleotide binding domain 2 | destabilising, favourable torsion angle of Gln residue, ∆∆G -0.21kcal/mol | destabilising -0.261kcal/mol | destabilising, -0.560kcal/mol | destabilising -0.194kcal/mol | increase in molecule flexibility  ΔΔSVib ENCoM: 0.076kcal/mol-1/K-1 | WT (3.5Å) to Mutant (3.5Å) | neutral effect (-0.55) |
| ABCB4 | Gallstone disease | G826R | Transmembrane domain 2 | destabilising, favourable torsion angle of Arg residue, ∆∆G -1.64kcal/mol | destabilising -0.904kcal/mol | destabilising, -2.250kcal/mol | destabilising -0.897kcal/mol | Decrease in molecule flexibility  ΔΔSVib ENCoM: -1.520kcal/mol-1/K-1 | WT (6.4Å) to Mutant (5.5Å) | deleterious effect (0.58) thermodynamic stability - destabilising thermal stability - destabilising |
| ABCB4 | Gallstone disease | R788L | Transmembrane domain 2 | stabilising, favourable torsion angle of Leu residue,  ∆∆G 0.21kcal/mol | destabilising -0.552kcal/mol | stabilising, 0.930kcal/mol | destabilising -1.115kcal/mol | increase in molecule flexibility  ΔΔSVib ENCoM: 0.137kcal/mol-1/K-1 | WT (5.8Å) to Mutant (5.9Å) | deleterious effect (0.57) thermodynamic stability - stabilising thermal stability - stabilising |
| ABCB4 | Gallstone disease | D686N | Unresolved 3D structure region |  |  |  |  |  |  |  |
| ABCB4 | Gallstone disease | M676I | Unresolved 3D structure region |  |  |  |  |  |  |  |
| ABCB4 | Gallstone disease | T651N | Unresolved 3D structure region |  |  |  |  |  |  |  |
| ABCB4 | Gallstone disease | K391E | Nucleotide binding domain 1 | stabilising, unfavourable torsion angle of Glu residue,  ∆∆G 0.61kcal/mol | stabilising, 0.634kcal/mol | stabilising, 0.500kcal/mol | stabilising, 0.244kcal/mol | increase in molecule flexibility  ΔΔSVib ENCoM: 0.135kcal/mol-1/K-1 | WT (3.4Å) to Mutant (3.6Å) | neutral effect (-0.40) |
| ABCB4 | Cholangiocarcinoma | Q668H | Unresolved 3D structure region |  |  |  |  |  |  |  |
| ABCB4 | . | Q1106H | Nucleotide binding domain 2 | destabilising, no change of torsion angle of His residue,  ∆∆G -2.16kcal/mol | destabilising -0.672kcal/mol | stabilising, -0.060kcal/mol | destabilising -0.863kcal/mol | increase in molecule flexibility  ΔΔSVib ENCoM: 0.412kcal/mol-1/K-1 | WT (3.8Å) to Mutant (4.0Å) | neutral effect (-0.24) |
| ABCB4 | . | F522S | Nucleotide binding domain 1 | destabilising, unfavourable torsion angle of Ser residue, ∆∆G -1.06kcal/mol | destabilising -1.486kcal/mol | destabilising, -1.190kcal/mol | destabilising -1.372kcal/mol | increase in molecule flexibility ΔΔSVib ENCoM:  1.320kcal/mol-1/K-1 | WT (4.2Å) to Mutant (3.4Å) | deleterious effect (0.54) thermodynamic stability - destabilising thermal stability - destabilising |
| ABCB4 | . | Y467D | Nucleotide binding domain 1 | destabilising, favourable torsion angle of Asp residue, ∆∆G -1.95kcal/mol | destabilising -1.536kcal/mol | destabilising -0.660kcal/mol | destabilising -1.731kcal/mol | increase in molecule flexibility ΔΔSVib ENCoM:  0.852kcal/mol-1/K-1 | WT (4.2Å) to Mutant (4.4Å) | deleterious effect (0.59) thermodynamic stability - destabilising thermal stability - destabilising |
| ABCB4 | . | G270R | Transmembrane domain 1 | destabilising, favourable torsion angle of Arg residue, ∆∆G -1.91kcal/mol | destabilising -1.296kcal/mol | destabilising -3.150kcal/mol | destabilising -1.045kcal/mol | decrease in molecule flexibility ΔΔSVib ENCoM:  -1.221kcal/mol-1/K-1 | WT (5.3Å) to Mutant (4.3Å) | deleterious effect (0.54) thermodynamic stability - destabilising thermal stability - destabilising |
| ABCB4 | . | G124C | Transmembrane domain 1 | stabilising,  unfavourable torsion angle of Cys residue, ∆∆G 0.66kcal/mol | destabilising -0.745kcal/mol | destabilising -0.560kcal/mol | destabilising -0.745kcal/mol | decrease in molecule flexibility ΔΔSVib ENCoM:  -0.736kcal/mol-1/K-1 | WT (6.0Å) to Mutant (6.3Å) | deleterious effect (0.60) thermodynamic stability - destabilising thermal stability - destabilising |
| ABCB4 | . | M113V | Transmembrane domain 1 | destabilising,  unfavourable torsion angle of Val residue, ∆∆G -2.9kcal/mol | destabilising -1.212kcal/mol | destabilising -1.060kcal/mol | destabilising -1.412kcal/mol | increase in molecule flexibility ΔΔSVib ENCoM:  0.432kcal/mol-1/K-1 | WT (7.5Å) to Mutant (8.1Å) | neutral effect (-0.11) |
| ABCB4 | . | L73V | Transmembrane domain 1 | destabilising,  unfavourable torsion angle of Val residue, ∆∆G -1.61kcal/mol | destabilising -0.478kcal/mol | destabilising -0.590kcal/mol | destabilising -0.709kcal/mol | increase in molecule flexibility ΔΔSVib ENCoM:  0.066kcal/mol-1/K-1 | WT (3.5Å) to Mutant (3.6Å) | neutral effect (-0.46) |
| ABCB4 | . | I1153K | Nucleotide binding domain 2 | stabilising,  favourable torsion angle of Lys residue, ∆∆G 2.69kcal/mol | destabilising -2.074kcal/mol | destabilising -2.210kcal/mol | destabilising -1.884kcal/mol | decrease in molecule flexibility ΔΔSVib ENCoM:  -0.039kcal/mol-1/K-1 | WT (7.4Å) to Mutant (7.6Å) | deleterious effect (0.62) thermodynamic stability - destabilising thermal stability - destabilising |
| ABCB4 | . | C1124R | Nucleotide binding domain 2 | stabilising,  unfavourable torsion angle of Arg residue, ∆∆G 3.44kcal/mol | stabilising, 0.051kcal/mol | stabilising, 0.010kcal/mol | destabilising -0.276kcal/mol | decrease in molecule flexibility ΔΔSVib ENCoM:  -0.074kcal/mol-1/K-1 | WT (3.7Å) to Mutant (4.1Å) | deleterious effect (0.82) thermodynamic stability - destabilising thermal stability - destabilising |
| ABCB4 | . | G1113R | Nucleotide binding domain 2 | stabilising,  favourable torsion angle of Arg residue, ∆∆G 0.52kcal/mol | destabilising -1.123kcal/mol | destabilising -2.140kcal/mol | destabilising -1.114kcal/mol | decrease in molecule flexibility ΔΔSVib ENCoM:  -1.012kcal/mol-1/K-1 | WT (9.8Å) to Mutant (8.9Å) | deleterious effect (0.66) thermodynamic stability - destabilising thermal stability - destabilising |
| ABCB4 | . | V1093M | Nucleotide binding domain 2 | destabilising,  favourable torsion angle of Met residue, ∆∆G -3.29kcal/mol | destabilising -0.963kcal/mol | destabilising -2.390kcal/mol | destabilising -0.586kcal/mol | decrease in molecule flexibility ΔΔSVib ENCoM:  -0.435kcal/mol-1/K-1 | WT (6.3Å) to Mutant (6.6Å) | deleterious effect (0.02) thermodynamic stability - destabilising thermal stability - destabilising |
| ABCB4 | . | A578S | Nucleotide binding domain 1 | destabilising,  unfavourable torsion angle of Ser residue, ∆∆G -2.82kcal/mol | destabilising -0.902kcal/mol | destabilising -1.980kcal/mol | destabilising -0.862kcal/mol | decrease in molecule flexibility ΔΔSVib ENCoM:  -0.277kcal/mol-1/K-1 | WT (6.2Å) to Mutant (6.1Å) | neutral effect (-0.22) |
| ABCB4 | . | V192I | Transmembrane domain 1 | stabilising,  unfavourable torsion angle of Ile residue, ∆∆G 1.12kcal/mol | stabilising, 0.713kcal/mol | destabilising -0.190kcal/mol | stabilising, 0.331kcal/mol | decrease in molecule flexibility ΔΔSVib ENCoM:  -0.123kcal/mol-1/K-1 | WT (5Å) to Mutant (5Å) | neutral effect (-0.44) |
| ABCB4 | . | R1224H | Nucleotide binding domain 2 | stabilising,  favourable torsion angle of His residue, ∆∆G 0.04kcal/mol | destabilising -1.706kcal/mol | destabilising -0.520kcal/mol | destabilising -1.473kcal/mol | decrease in molecule flexibility ΔΔSVib ENCoM:  -0.217kcal/mol-1/K-1 | WT (5.3Å) to Mutant (5.1Å) | deleterious effect (0.13) thermodynamic stability - destabilising thermal stability - destabilising |
| ABCB4 | . | D804N | Transmembrane domain 2 | destabilising,  favourable torsion angle of Asn residue,  ∆∆G -0.79kcal/mol | destabilising -0.078kcal/mol | stabilising, 0.410kcal/mol | destabilising -0.389kcal/mol | increase in molecule flexibility ΔΔSVib ENCoM:  0.074kcal/mol-1/K-1 |  | neutral effect (-0.12) |
| ABCB11 | ICP | ABCB11 D1284N | Nucleotide binding domain 2 | destabilising, favourable torsion angle of Asn residue,  ∆∆G -1.32kcal/mol | stabilising 0.035kcal/mol | stabilising 0.150kcal/mol | destabilising -0.231kcal/mol | decrease in molecule flexibility  ΔΔSVib ENCoM:  -0.190kcal/mol-1/K-1 | WT (4.2Å) to Mutant (4.3Å) | deleterious effect (0.13) thermodynamic stability - destabilising thermal stability - stabilising |
| ABCB11 | ICP | ABCB11 R1050H | Nucleotide binding domain 2 | destabilising, unfavourable torsion angle of His residue,  ∆∆G -0.57kcal/mol | destabilising -1.256kcal/mol | stabilising 0.045kcal/mol | destabilising -1.568kcal/mol | increase in molecule flexibility  ΔΔSVib ENCoM:  0.148kcal/mol-1/K-1 | WT (4.2Å) to Mutant (4.5Å) | neutral effect (-0.62) |
| ABCB11 | ICP | M677V | Unresolved 3D structure region |  |  |  |  |  |  |  |
| ABCB11 | ICP | N591S | Nucleotide binding domain 1 | destabilising, favourable torsion angle of Ser residue,  ∆∆G -1.52kcal/mol | stabilising 0.157kcal/mol | destabilising -0.800kcal/mol | destabilising -0.009kcal/mol | increase in molecule flexibility  ΔΔSVib ENCoM:  0.002kcal/mol-1/K-1 | WT (3.2Å) to Mutant (3.1Å) | neutral effect (-0.50) |
| ABCB11 | ICP | ABCB11 V284A | Transmembrane domain 1 | destabilising, unfavourable torsion angle of Ala residue,  ∆∆G -2.32kcal/mol | stabilising 2.154kcal/mol | destabilising -1.080kcal/mol | destabilising -1.937kcal/mol | increase in molecule flexibility  ΔΔSVib ENCoM:  0.660kcal/mol-1/K-1 | WT (8.4Å) to Mutant (8.5Å) | deleterious effect (0.14) thermodynamic stability - destabilising thermal stability - destabilising |
| ABCB11 | Gallstone disease | A1260P | Nucleotide binding domain 2 | destabilising, unfavourable torsion angle of Pro residue,  ∆∆G -3.32kcal/mol | destabilising -1.328kcal/mol | destabilising -4.450kcal/mol | destabilising -0.651kcal/mol | decrease in molecule flexibility  ΔΔSVib ENCoM:  -0.279kcal/mol-1/K-1 | WT (7.4Å) to Mutant (6.3Å) | deleterious effect (0.46) thermodynamic stability - destabilising thermal stability - destabilising |
| ABCB11 | Gallstone disease | Q976R | Transmembrane domain 2 | destabilising, favourable torsion angle of Arg residue,  ∆∆G -0.33kcal/mol | stabilising 0.240kcal/mol | stabilising 0.380kcal/mol | destabilising -0.098kcal/mol | increase in molecule flexibility  ΔΔSVib ENCoM:  0.150kcal/mol-1/K-1 | WT (3.6Å) to Mutant (3.6Å) | neutral effect (-0.58) |
| ABCB11 | Gallstone disease | A926S | Transmembrane domain 2 | stabilising, favourable torsion angle of Ser residue,  ∆∆G 0.73kcal/mol | destabilising -0.573kcal/mol | destabilising -2.040kcal/mol | destabilising -0.568kcal/mol | decrease in molecule flexibility  ΔΔSVib ENCoM:  -0.076kcal/mol-1/K-1 | WT (3.1Å) to Mutant (3.2Å) | neutral effect (-0.58) |
| ABCB11 | Gallstone disease | A679V | Unresolved 3D structure region |  |  |  |  |  |  |  |
| ABCB11 | Gallstone disease | N539D | Nucleotide binding domain 1 | destabilising, favourable torsion angle of Asp residue,  ∆∆G -0.13kcal/mol | stabilising 0.215kcal/mol | stabilising 0.350kcal/mol | destabilising -0.142kcal/mol | increase in molecule flexibility  ΔΔSVib ENCoM:  0.066kcal/mol-1/K-1 | WT (3.4Å) to Mutant (3.4Å) | neutral effect (-0.51) |
| ABCB11 | Gallstone disease | R487C | Nucleotide binding domain 1 | stabilising, favourable torsion angle of Cys residue,  ∆∆G 2.17kcal/mol | destabilising -0.413kcal/mol | destabilising -0.130kcal/mol | destabilising -0.432kcal/mol | increase in molecule flexibility  ΔΔSVib ENCoM:  0.297kcal/mol-1/K-1 | WT (3.6Å) to Mutant (3.6Å) | deleterious effect (0.44) thermodynamic stability - destabilising thermal stability - destabilising |
| ABCB11 | Gallstone disease | A311T | Transmembrane domain 1 | destabilising, favourable torsion angle of Thr residue,  ∆∆G -0.87kcal/mol | destabilising -1.905kcal/mol | destabilising -2.540kcal/mol | destabilising -1.769kcal/mol | decrease in molecule flexibility  ΔΔSVib ENCoM:  -0.156kcal/mol-1/K-1 | WT (7.7Å) to Mutant (8.6Å) | neutral effect (-0.07) |
| ABCB11 | Gallstone disease | V95I | Transmembrane domain 1 | destabilising, unfavourable torsion angle of Ile residue,  ∆∆G -0.27kcal/mol | destabilising -0.172kcal/mol | stabilising 0.360kcal/mol | destabilising -0.647kcal/mol | decrease in molecule flexibility  ΔΔSVib ENCoM:  -0.250kcal/mol-1/K-1 | WT (3.6Å) to Mutant (3.8Å) | neutral effect (-0.67) |
| ABCB11 | Gallstone disease | D94N | Transmembrane domain 1 | destabilising, unfavourable torsion angle of Asn residue,  ∆∆G -1.59kcal/mol | destabilising -0.477kcal/mol | stabilising 0.570kcal/mol | destabilising -0.884kcal/mol | increase in molecule flexibility  ΔΔSVib ENCoM:  0.180kcal/mol-1/K-1 | WT (5.3Å) to Mutant (6.0Å) | neutral effect (-0.03) |
| ABCB11 | Gallstone disease | K12R | Unresolved 3D structure region |  |  |  |  |  |  |  |
| ABCB11 | . | R1268Q | Nucleotide binding domain 2 | destabilising, unfavourable torsion angle of Gln residue,  ∆∆G -1.21kcal/mol | destabilising -0.578kcal/mol | destabilising -0.750kcal/mol | destabilising -0.614kcal/mol | increase in molecule flexibility  ΔΔSVib ENCoM:  0.478kcal/mol-1/K-1 | WT (4.4Å) to Mutant (4.5Å) | deleterious effect (0.01) thermodynamic stability - destabilising thermal stability - destabilising |
| ABCB11 | . | R1153H | Nucleotide binding domain 2 | destabilising, unfavourable torsion angle of His residue,  ∆∆G -1.21kcal/mol | destabilising -2.355kcal/mol | destabilising -0.560kcal/mol | destabilising -2.156kcal/mol | increase in molecule flexibility  ΔΔSVib ENCoM:  0.014kcal/mol-1/K-1 | WT (5.3Å) to Mutant (5.4Å) | deleterious effect (0.10) thermodynamic stability - destabilising thermal stability - destabilising |
| ABCB11 | . | R1050C | Nucleotide binding domain 2 | destabilising, unfavourable torsion angle of Cys residue,  ∆∆G -0.06kcal/mol | destabilising -0.986kcal/mol | destabilising -0.660kcal/mol | destabilising -0.988kcal/mol | increase in molecule flexibility  ΔΔSVib ENCoM:  0.227kcal/mol-1/K-1 | WT (4.2Å) to Mutant (4.2Å) | deleterious effect (0.16) thermodynamic stability - destabilising thermal stability - destabilising |
| ABCB11 | . | A995V | Transmembrane domain 2 | stabilising, unfavourable torsion angle of Val residue,  ∆∆G 1.74kcal/mol | stabilising 0.474kcal/mol | destabilising -1.030kcal/mol | stabilising 0.291kcal/mol | decrease in molecule flexibility  ΔΔSVib ENCoM:  -0.389kcal/mol-1/K-1 | WT (7.9Å) to Mutant (8.5Å) | deleterious effect (0.19) thermodynamic stability - destabilising thermal stability - destabilising |
| ABCB11 | . | R616H | Nucleotide binding domain 1 | destabilising, unfavourable torsion angle of His residue,  ∆∆G -1.93kcal/mol | destabilising -1.069kcal/mol | stabilising 0.130kcal/mol | destabilising -1.056kcal/mol | decrease in molecule flexibility  ΔΔSVib ENCoM:  -0.977kcal/mol-1/K-1 | WT (3.6Å) to Mutant (3.5Å) | neutral effect (-0.39) |
| ABCB11 | . | T463I | Nucleotide binding domain 1 | stabilising, favourable torsion angle of Ile residue,  ∆∆G 4.64kcal/mol | stabilising 0.642kcal/mol | stabilising 1.030kcal/mol | stabilising 0.054kcal/mol | decrease in molecule flexibility  ΔΔSVib ENCoM:  -0.002kcal/mol-1/K-1 | WT (6.9Å) to Mutant (6.6Å) | deleterious effect (0.51) thermodynamic stability - destabilising thermal stability - destabilising |
| ABCB11 | . | R1235Q | Nucleotide binding domain 2 | destabilising, favourable torsion angle of Gln residue,  ∆∆G -0.69kcal/mol | destabilising -0.920kcal/mol | destabilising -0.750kcal/mol | destabilising -0.809kcal/mol | increase in molecule flexibility  ΔΔSVib ENCoM:  1.025kcal/mol-1/K-1 | WT (5.5Å) to Mutant (4.6Å) | neutral effect (-0.07) |
| ABCB11 | . | D1172G | Nucleotide binding domain 2 | destabilising, unfavourable torsion angle of Arg residue,  ∆∆G -3.01kcal/mol | destabilising -0.260kcal/mol | stabilising 0.020kcal/mol | destabilising -0.553kcal/mol | increase in molecule flexibility  ΔΔSVib ENCoM:  0.277kcal/mol-1/K-1 | WT (3.8Å) to Mutant (4.3Å) | neutral effect (-0.22) |
| ABCB11 | . | S488C | Nucleotide binding domain 1 | stabilising, unfavourable torsion angle of Cys residue,  ∆∆G 1.72kcal/mol | destabilising -0.002kcal/mol | stabilising 1.200kcal/mol | destabilising -0.392kcal/mol | increase in molecule flexibility  ΔΔSVib ENCoM:  0.012kcal/mol-1/K-1 | WT (3.1Å) to Mutant (3.2Å) | neutral effect (-0.15) |
| ABCB11 | . | N116S | Transmembrane domain 1 | stabilising, unfavourable torsion angle of Ser residue,  ∆∆G 1.12kcal/mol | destabilising -0.471kcal/mol | destabilising -1.620kcal/mol | destabilising -0.252kcal/mol | increase in molecule flexibility  ΔΔSVib ENCoM:  0.000kcal/mol-1/K-1 | WT (4.3Å) to Mutant (4.4Å) | neutral effect (-0.03) |
| ABCB11 | . | P77L | Transmembrane domain 1 | destabilising, unfavourable torsion angle of Leu residue,  ∆∆G -4.79kcal/mol | stabilising 0.042kcal/mol | stabilising 2.790kcal/mol | destabilising -0.860kcal/mol | decrease in molecule flexibility  ΔΔSVib ENCoM:  -0.928kcal/mol-1/K-1 | WT (7.3Å) to Mutant (7.3Å) | deleterious effect (0.93) thermodynamic stability - stabilising thermal stability - destabilising |
| ABCB11 | . | V1212I | Nucleotide binding domain 2 | destabilising, unfavourable torsion angle of Ile residue,  ∆∆G -2.73kcal/mol | destabilising -0.367kcal/mol | destabilising -0.180kcal/mol | destabilising -0.631kcal/mol | decrease in molecule flexibility  ΔΔSVib ENCoM:  -0.465kcal/mol-1/K-1 | WT (6.2Å) to Mutant (5.9Å) | neutral effect (-0.15) |
| ABCB11 | . | F651S | Nucleotide binding domain 1 | destabilising, favourable torsion angle of Ser residue,  ∆∆G -0.27kcal/mol | destabilising -2.245kcal/mol | destabilising -1.940kcal/mol | destabilising -2.011kcal/mol | increase in molecule flexibility  ΔΔSVib ENCoM:  0.758kcal/mol-1/K-1 | WT (4.1Å) to Mutant (3.9Å) | deleterious effect (0.33) thermodynamic stability - destabilising thermal stability - destabilising |
| ABCB11 | . | I245F | Transmembrane domain 1 | stabilising, unfavourable torsion angle of Phe residue,  ∆∆G 0.39kcal/mol | destabilising -1.160kcal/mol | destabilising -0.210kcal/mol | destabilising -1.121kcal/mol | decrease in molecule flexibility  ΔΔSVib ENCoM:  -0.461kcal/mol-1/K-1 | WT (7.0Å) to Mutant (6.9Å) | deleterious effect (0.02) thermodynamic stability - destabilising thermal stability - destabilising |
| ABCB11 | . | D191A | Transmembrane domain 1 | destabilising, favourable torsion angle of Ala residue,  ∆∆G -0.55kcal/mol | stabilising 0.089kcal/mol | stabilising 1.300kcal/mol | destabilising -0.495kcal/mol | increase in molecule flexibility  ΔΔSVib ENCoM:  0.395kcal/mol-1/K-1 | WT (4.0Å) to Mutant (3.9Å) | deleterious effect (0.16) thermodynamic stability - destabilising thermal stability - destabilising |
| ATP8B1 | ICP | R384H | Transmembrane domain 1 | stabilising, unfavourable torsion angle of His residue,  ∆∆G 0.54kcal/mol | destabilising -1.573kcal/mol | stabilising 0.49kcal/mol | destabilising -1.915kcal/mol | decrease in molecule flexibility  ΔΔSVib ENCoM:  -0.91kcal/mol-1/K-1 | WT (4.1Å) to Mutant (4.5Å) | neutral effect (-0.29) |
| ATP8B1 | Gallstone disease | V1161A | Transmembrane domain 2 | destabilising, favourable torsion angle of Ala residue,  ∆∆G -3.54kcal/mol | destabilising -1.436kcal/mol | destabilising -0.25kcal/mol | destabilising -1.667kcal/mol | decrease in molecule flexibility  ΔΔSVib ENCoM:  -0.82kcal/mol-1/K-1 | WT (3.4Å) to Mutant (3.2Å) | deleterious effect (0.18) thermodynamic stability - destabilising thermal stability - destabilising |
| ATP8B1 | Gallstone disease | T1092I | Transmembrane domain 2 | destabilising, unfavourable torsion angle of Ile residue,  ∆∆G -2.54kcal/mol | stabilising 0.358kcal/mol | stabilising 1.04kcal/mol | destabilising -0.06kcal/mol | increase in molecule flexibility  ΔΔSVib ENCoM:  0.24kcal/mol-1/K-1 | WT (6.9Å) to Mutant (6.7Å) | deleterious effect (0.41) thermodynamic stability - destabilising thermal stability - destabilising |
| ATP8B1 | Gallstone disease | M674T | Nucleotide binding domain | destabilising, unfavourable torsion angle of Thr residue,  ∆∆G -0.18kcal/mol | stabilising 0.267kcal/mol | destabilising -1.0kcal/mol | destabilising -0.097kcal/mol | increase in molecule flexibility  ΔΔSVib ENCoM:  0.1kcal/mol-1/K-1 | WT (3.3Å) to Mutant (3.2Å) | neutral effect (-0.55) |
| ATP8B1 | Gallstone disease | I577V | Nucleotide binding domain | destabilising, favourable torsion angle of Val residue,  ∆∆G -0.8kcal/mol | destabilising -1.606kcal/mol | destabilising -2.38kcal/mol | destabilising -1.321kcal/mol | decrease in molecule flexibility  ΔΔSVib ENCoM:  -0.88kcal/mol-1/K-1 | WT (9.2Å) to Mutant (9.1Å) | neutral effect (-0.34) |
| ATP8B1 | Gallstone disease | H78Q | Transmembrane domain 1 |  |  |  |  |  |  |  |
| ATP8B1 | Gallstone disease | D14Y | Transmembrane domain 1 |  |  |  |  |  |  |  |
| ATP8B1 | Cirrhosis | I513T | Nucleotide binding domain | destabilising, unfavourable torsion angle of Thr residue,  ∆∆G -2.26kcal/mol | destabilising -3.232kcal/mol | destabilising -2.18kcal/mol | destabilising -3.088cal/mol | decrease in molecule flexibility  ΔΔSVib ENCoM:  -2.98kcal/mol-1/K-1 | WT (5.5Å) to Mutant (4.8Å) | deleterious effect (0.01) thermodynamic stability - destabilising thermal stability - destabilising |
| ATP8B1 | Cirrhosis, secondary malignant neoplasm of liver and bile duct, gallstone disease | D70N | Transmembrane domain 1 | destabilising, favourable torsion angle of Asn residue,  ∆∆G -0.61kcal/mol | stabilising 0.505kcal/mol | stabilising 0.01kcal/mol | stabilising 0.385kcal/mol | increase in molecule flexibility  ΔΔSVib ENCoM:  0.37kcal/mol-1/K-1 | WT (3.4Å) to Mutant (3.5Å) | neutral effect (-0.44) |
| ATP8B1 | . | G308S | Actuator domain | destabilising, favourable torsion angle of Ser residue,  ∆∆G -2.09kcal/mol | destabilising -1.593kcal/mol | destabilising -2.5kcal/mol | destabilising -1.712kcal/mol | decrease in molecule flexibility  ΔΔSVib ENCoM:  -1.4kcal/mol-1/K-1 | WT (12.6Å) to Mutant (12.1Å) | deleterious effect (0.30) thermodynamic stability - destabilising thermal stability - destabilising |
| ATP8B1 | . | G308A | Actuator domain 1 | destabilising, unfavourable torsion angle of Ala residue,  ∆∆G -1.57kcal/mol | destabilising -0.215kcal/mol | destabilising -0.47kcal/mol | destabilising -0.51kcal/mol | decrease in molecule flexibility  ΔΔSVib ENCoM:  -0.09kcal/mol-1/K-1 | WT (12.6Å) to Mutant (12.4Å) | deleterious effect (0.23) thermodynamic stability - destabilising thermal stability - destabilising |
| ATP8B1 | . | R628Q | Nucleotide binding domain | stabilising, favourable torsion angle of Gln residue,  ∆∆G 0.28kcal/mol | destabilising -0.805kcal/mol | destabilising -0.76kcal/mol | destabilising -0.69kcal/mol | decrease in molecule flexibility  ΔΔSVib ENCoM:  -0.88kcal/mol-1/K-1 | WT (4.5Å) to Mutant (4.5Å) | neutral effect (-0.18) |
| NR1H4 | . | N358H | Ligand-binding domain | stabilising, favourable torsion angle of His residue,  ∆∆G 0.75kcal/mol | destabilising -0.758kcal/mol | stabilising 0.050kcal/mol | destabilising -0.752kcal/mol | decrease in molecule flexibility  ΔΔSVib ENCoM:  -0.157kcal/mol-1/K-1 | WT (3.1Å) to Mutant (3.3Å) | neutral effect (-0.67) |
| NR1H4 | . | M173T | Unresolved 3D structure region |  |  |  |  |  |  |  |

**Supplementary Figure legends**

**Supplementary Figure 1.**

**Flow diagram illustrating the patient inclusion for the phenotype to genotype analysis.** This diagram illustrates the number of volunteers included with a phenotypic presentation of intrahepatic cholestasis of pregnancy selected from the 5236 exome sequencing data available in the Genes and Health database.

**Supplementary Figure 2.**

**NR1H4 variant summary in a 2-dimensional illustration.** NR1H4 also known as FXR is a nuclear receptor within hepatocytes. Variants are divided into their phenotypic presentation: No phenotype previously reported, and intrahepatic cholestasis of pregnancy. Bold border represents variants that are unique to the Genes & Health cohort.

**Supplementary Figure 3.**

**Overall summary of all variants included for the protein modelling in the Genes and Health cohort for all five gene candidates.** All variants included for the analysis were modelled and stratified by their predicted structural effect on the 3D structure. *TJP2 structure not included as no 3D structure available.

**Supplementary Figure 4.**

**Rare variants at the energy transduction interface between the ligand binding and ATP catalytic domains of ABCB4 and ABCB11**

**a. Topological cartoon showing the organisation of the transmembrane domains (TMDs) and the interaction with the nucleotide binding domains (NBDs) of ABCB4 and ABCB11.** Each TMD comprises 6 transmembrane alpha helices (TMH 1-6 of TMD1 in rainbow colouring, TMH 7-12 of TMD2 in grey). The intracellular loops between TMH 2 and 3, 4 and 5, 8 and 9, and 10 and 11 extend into the cytosol to interact with the NBDs (blue and pink squares represent NBD1 and NBD2 respectively). At the base of each of these intracellular loops is a coupling helix (labelled CH1-4) which interdigitate with grooves on the top surface of the NBDs. The coupling helices interact with the surface of the NBDs and are assumed to convey conformational changes in a bidirectional manner: to the NBDs from the TMDs to signal substrate binding to the latter and, vice versa to the TMDs from the NBDs to drive the conformational changes associated with ATP binding (resulting in substrate efflux) and the conformational changes associated with ATP hydrolysis to reset the transporter for binding of the next transport substrate. The domain colour coding is preserved in 4B and 4C.

**b. ABCB4 variant Q1106H is in a functionally sensitive location for energy transduction, disrupting the interaction between CH2 and NBD2.** Left panel; cartoon representation of ABCB4 with bound PC. PC is shown in sphere format and coloured elementally (pdb: 7NIV). Q1106 also shown as spheres with elemental colouring is located in a groove on the top surface of NBD2 (pink). The groove is occupied by CH2 formed between TMH4 (yellow) and TMH5 (orange). The distance between the side chain nitrogen (blue) of Q1106 and the nearest atom in the coupling helix, the oxygen (red) of the peptide bond formed by G270 is indicated in the zoomed image where the relevant amino acids are shown in stick format with elemental colouring (nitrogen in blue, oxygen in red, carbon in green). Right panel; cartoon representation of ABCB4 with 2 x Mg^2+^ and 2 x ATP (pdb: 6S7P). Q1106 is now in close proximity to Q272 in CH2. The distance between the side chain nitrogen (blue) of Q1106 and the oxygen (red) of the Q272 is indicated in the zoomed image. Interatom distances were measured in Pymol.

**c. ABCB11 variant D191A is in a functionally sensitive location for energy transduction, disrupting the interaction between CH1 and NBD1.** Left panel; cartoon representation of ABCB11 (pdb: 6LR0). D191 which is shown as spheres with elemental colouring is located in CH1 where it interacts with both Y472 on the top surface of NBD1 (blue) and R946 from CH4. Minimum distances between the oxygens of the carboxylic acid side chain of D191 and the oxygen of Y472 and the nitrogen of the guanidinium group of R946 are shown in the zoomed image where the relevant amino acids are shown in stick format with elemental colouring as above.

**Supplementary Figure 1.
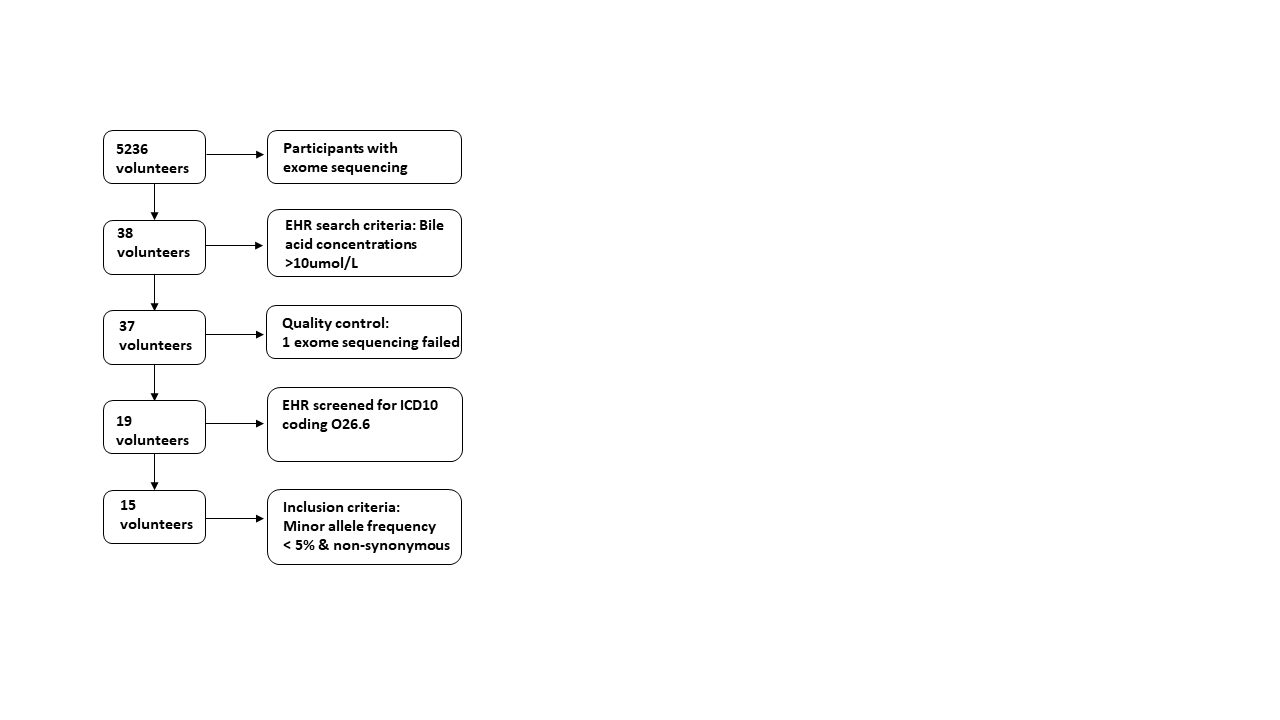
**

**Supplementary Figure 2.**

**
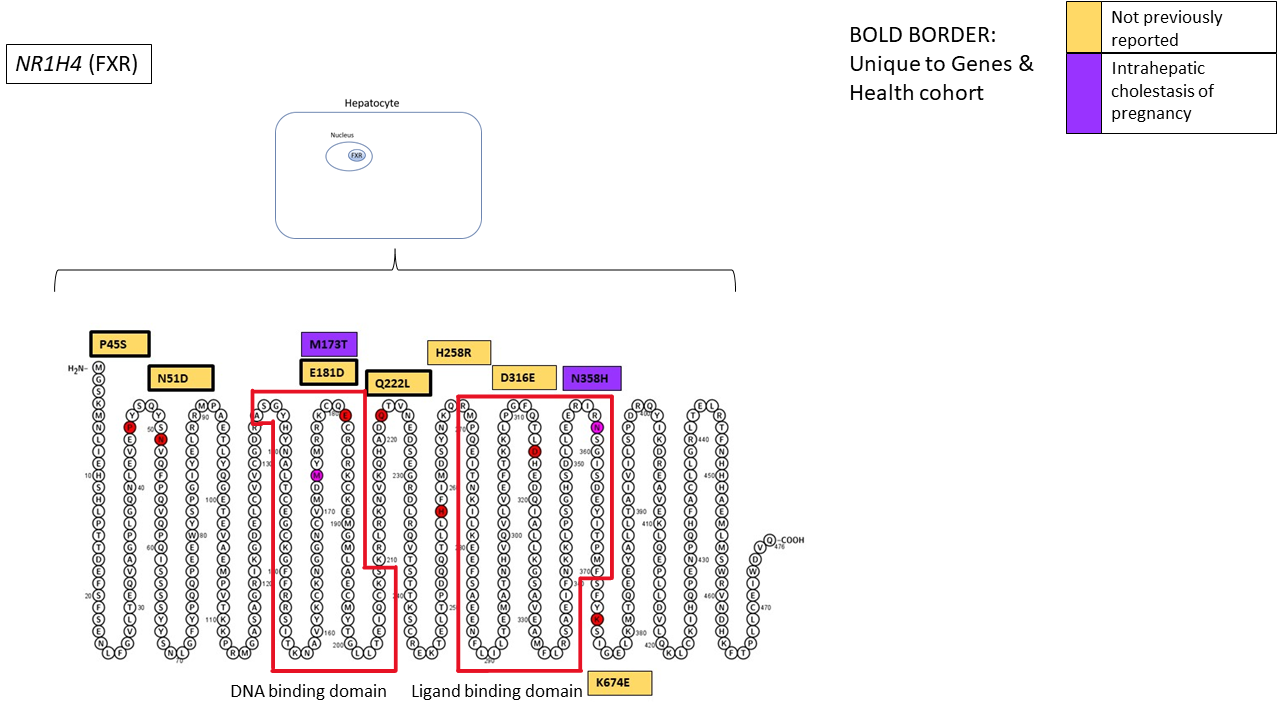
**

**Supplementary Figure 3.
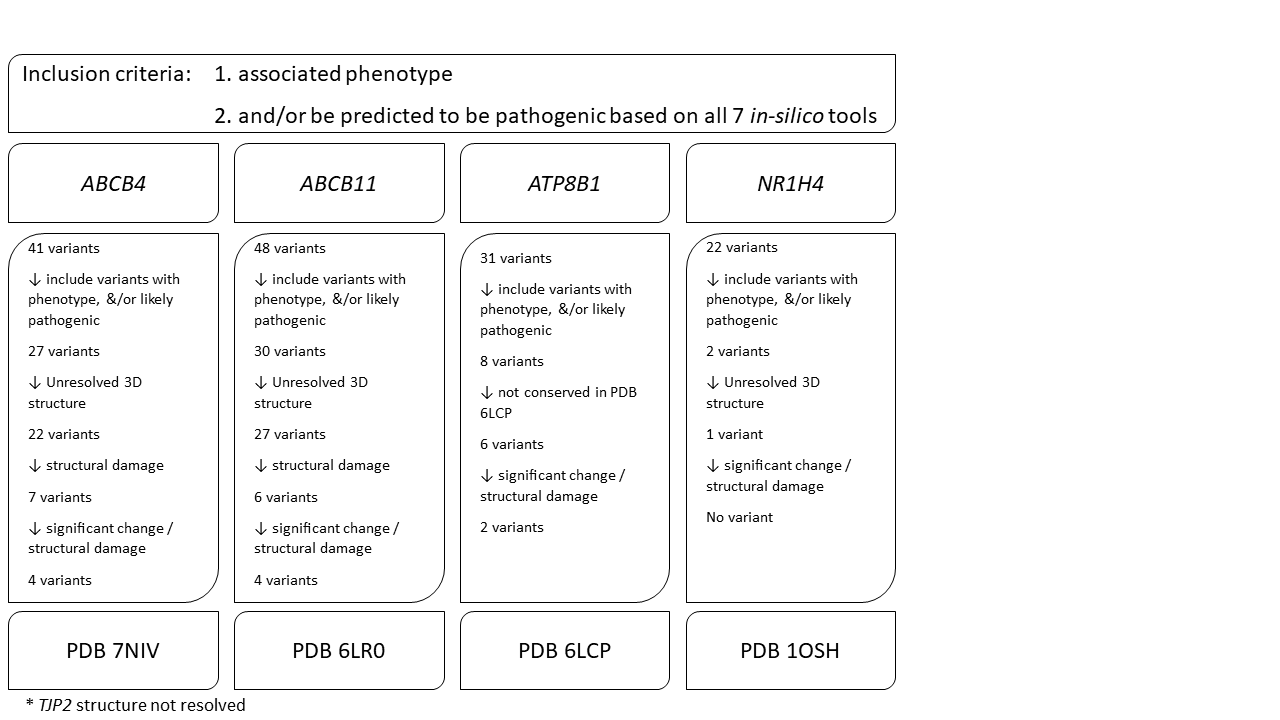
**

**Supplementary Figure 4. Rare variants at the energy transduction interface between the ligand binding and ATP catalytic domains of ABCB4 and ABCB11**

**
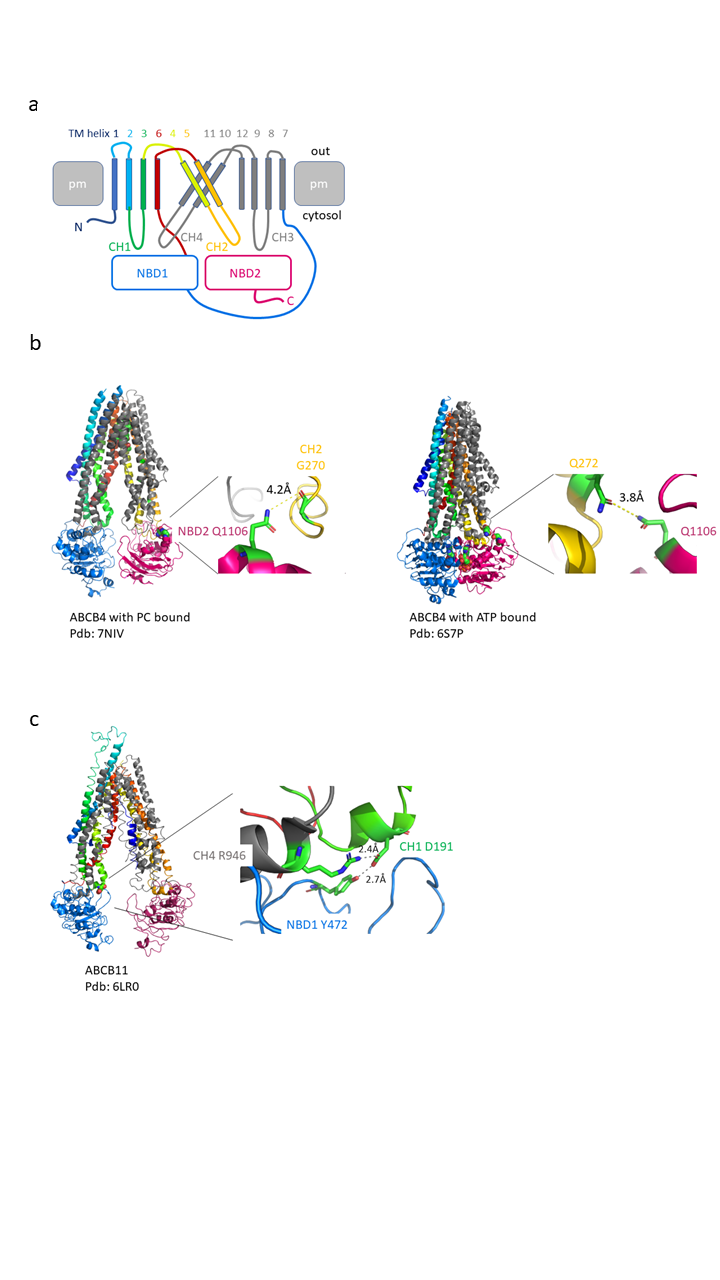
**

**References**

1. Nakken, K.E., K.J. Labori, O.K. Rødningen, et al., *ABCB4 sequence variations in young adults with cholesterol gallstone disease.* Liver International, 2009. **29**(5): p. 743-747.

2. Delaunay, J.-L., A.-M. Durand-Schneider, C. Dossier, et al., *A functional classification of ABCB4 variations causing progressive familial intrahepatic cholestasis type 3.* Hepatology, 2016. **63**(5): p. 1620-1631.

3. Dixon, P.H., M. Sambrotta, J. Chambers, et al., *An expanded role for heterozygous mutations of ABCB4, ABCB11, ATP8B1, ABCC2 and TJP2 in intrahepatic cholestasis of pregnancy.* Scientific Reports, 2017. **7**(1): p. 11823.

4. Bacq, Y., C. Gendrot, F. Perrotin, et al., *ABCB4 gene mutations and single-nucleotide polymorphisms in women with intrahepatic cholestasis of pregnancy.* J Med Genet, 2009. **46**(10): p. 711-5.

5. Degiorgio, D., C. Colombo, M. Seia, et al., *Molecular characterization and structural implications of 25 new ABCB4 mutations in progressive familial intrahepatic cholestasis type 3 (PFIC3).* European Journal of Human Genetics, 2007. **15**(12): p. 1230-1238.

6. Wendum, D., V. Barbu, O. Rosmorduc, et al., *Aspects of liver pathology in adult patients with MDR3/ABCB4 gene mutations.* Virchows Archiv, 2012. **460**(3): p. 291-298.

7. Vitale, G., S. Gitto, F. Raimondi, et al., *Cryptogenic cholestasis in young and adults: ATP8B1, ABCB11, ABCB4, and TJP2 gene variants analysis by high-throughput sequencing.* Journal of Gastroenterology, 2018. **53**(8): p. 945-958.

8. Poupon, R., L. Arrive, and O. Rosmorduc, *The cholangiographic features of severe forms of ABCB4/MDR3 deficiency-associated cholangiopathy in adults.* Gastroentérologie Clinique et Biologique, 2010. **34**(6): p. 380-387.

9. Pauli-Magnus, C., T. Lang, Y. Meier, et al., *Sequence analysis of bile salt export pump (ABCB11) and multidrug resistance p-glycoprotein 3 (ABCB4, MDR3) in patients with intrahepatic cholestasis of pregnancy.* Pharmacogenetics and Genomics, 2004. **14**(2).

10. Aydın, G.A., G. Özgen, and O. Görükmez, *The role of genetic mutations in intrahepatic cholestasis of pregnancy.* Taiwanese Journal of Obstetrics and Gynecology, 2020. **59**(5): p. 706-710.

11. Degiorgio, D., A. Crosignani, C. Colombo, et al., *ABCB4 mutations in adult patients with cholestatic liver disease: impact and phenotypic expression.* Journal of Gastroenterology, 2016. **51**(3): p. 271-280.

12. Poupon, R., O. Rosmorduc, P.Y. Boëlle, et al., *Genotype-phenotype relationships in the low-phospholipid-associated cholelithiasis syndrome: A study of 156 consecutive patients.* Hepatology, 2013. **58**(3): p. 1105-1110.

13. Schatz, S.B., C. Jüngst, V. Keitel-Anselmo, et al., *Phenotypic spectrum and diagnostic pitfalls of ABCB4 deficiency depending on age of onset.* Hepatology Communications, 2018. **2**(5): p. 504-514.

14. Tavian, D., D. Degiorgio, N. Roncaglia, et al., *A new splicing site mutation of the ABCB4 gene in intrahepatic cholestasis of pregnancy with raised serum γ-GT.* Digestive and Liver Disease, 2009. **41**(9): p. 671-675.

15. Huynh, M.T., T.T. Nguyen, S. Grison, et al., *Clinical characteristics and genetic profiles of young and adult patients with cholestatic liver disease.* Rev Esp Enferm Dig, 2019. **111**(10): p. 775-788.

16. Floreani, A., I. Carderi, D. Paternoster, et al., *Hepatobiliary phospholipid transporter ABCB4, MDR3 gene variants in a large cohort of Italian women with intrahepatic cholestasis of pregnancy.* Digestive and Liver Disease, 2008. **40**(5): p. 366-370.

17. Lang, T., M. Haberl, D. Jung, et al., *Genetic Variability, Haplotype Structures, and Ethnic Diversity of Hepatic Transporters MDR3 (ABCB4) and Bile Salt Export Pump (ABCB11).* Drug Metabolism and Disposition, 2006. **34**(9): p. 1582-1599.

18. Avena, A., S. Puggelli, M. Morris, et al., *ABCB4 variants in adult patients with cholestatic disease are frequent and underdiagnosed.* Digestive and Liver Disease, 2021. **53**(3): p. 329-344.

19. Shagrani, M., J. Burkholder, D. Broering, et al., *Genetic profiling of children with advanced cholestatic liver disease.* Clinical Genetics, 2017. **92**(1): p. 52-61.

20. Anzivino, C., M.R. Odoardi, E. Meschiari, et al., *ABCB4 and ABCB11 mutations in intrahepatic cholestasis of pregnancy in an Italian population.* Dig Liver Dis, 2013. **45**(3): p. 226-32.

21. Pauli-Magnus, C., R. Kerb, K. Fattinger, et al., *BSEP and MDR3 haplotype structure in healthy Caucasians, primary biliary cirrhosis and primary sclerosing cholangitis.* Hepatology, 2004. **39**(3): p. 779-791.

22. Colombo, C., P. Vajro, D. Degiorgio, et al., *Clinical features and genotype-phenotype correlations in children with progressive familial intrahepatic cholestasis type 3 related to ABCB4 mutations.* J Pediatr Gastroenterol Nutr, 2011. **52**(1): p. 73-83.

23. Kagawa, T., N. Watanabe, K. Mochizuki, et al., *Phenotypic differences in PFIC2 and BRIC2 correlate with protein stability of mutant Bsep and impaired taurocholate secretion in MDCK II cells.* American Journal of Physiology-Gastrointestinal and Liver Physiology, 2008. **294**(1): p. G58-G67.

24. Wang, L., C.J. Soroka, and J.L. Boyer, *The role of bile salt export pump mutations in progressive familial intrahepatic cholestasis type II.* J Clin Invest, 2002. **110**(7): p. 965-72.

25. Byrne, J.A., S.S. Strautnieks, G. Ihrke, et al., *Missense mutations and single nucleotide polymorphisms in ABCB11 impair bile salt export pump processing and function or disrupt pre-messenger RNA splicing.* Hepatology, 2009. **49**(2): p. 553-567.

26. Strautnieks, S.S., J.A. Byrne, L. Pawlikowska, et al., *Severe Bile Salt Export Pump Deficiency: 82 Different ABCB11 Mutations in 109 Families.* Gastroenterology, 2008. **134**(4): p. 1203-1214.e8.

27. Giovannoni, I., F. Callea, E. Bellacchio, et al., *Genetics and Molecular Modeling of New Mutations of Familial Intrahepatic Cholestasis in a Single Italian Center.* PLoS One, 2015. **10**(12): p. e0145021.

28. Liu, T., R.-X. Wang, J. Han, et al., *Changes in plasma bile acid profiles after partial internal biliary diversion in PFIC2 patients.* Annals of Translational Medicine, 2020. **8**(5): p. 185.

29. Wang, N.-L., Y.-L. Qiu, W.-C. Guan, et al., *Splicing analysis of rare/novel synonymous or intronic variants identified in ABCB11 heterozygotes presenting as progressive intrahepatic cholestasis with low γ-glutamyltransferase.* Hepatology Research, 2018. **48**(7): p. 574-584.

30. McKay, K., C. Bruce, J. Hartley, et al., *Mutation detection in cholestatic patients using microarray resequencing of ATP8B1 and ABCB11 [version 2; peer review: 2 approved, 1 approved with reservations].* F1000Research, 2013. **2**(32).

31. Fotoulaki, M., S. Giza, M. Jirsa, et al., *Beyond an Obvious Cause of Cholestasis in a Toddler: Compound Heterozygosity for ABCB11 Mutations.* Pediatrics, 2019. **143**(5).

32. Lam, P., C.L. Pearson, C.J. Soroka, et al., *Levels of plasma membrane expression in progressive and benign mutations of the bile salt export pump (Bsep/Abcb11) correlate with severity of cholestatic diseases.* American Journal of Physiology-Cell Physiology, 2007. **293**(5): p. C1709-C1716.

33. Zellos, A., L. Lykopoulou, A. Polydorou, et al., *Nasobiliary Drainage in an Episode of Intrahepatic Cholestasis in a Child With Mild ABCB11 Disease.* Journal of Pediatric Gastroenterology and Nutrition, 2012. **55**(1).

34. van Mil, S.W.C., W.L. van der Woerd, G. van der Brugge, et al., *Benign recurrent intrahepatic cholestasis type 2 is caused by mutations in ABCB11.* Gastroenterology, 2004. **127**(2): p. 379-384.

35. Knisely, A.S., S.S. Strautnieks, Y. Meier, et al., *Hepatocellular carcinoma in ten children under five years of age with bile salt export pump deficiency.* Hepatology, 2006. **44**(2): p. 478-486.

36. Slavetinsky, C. and E. Sturm, *Odevixibat and partial external biliary diversion showed equal improvement of cholestasis in a patient with progressive familial intrahepatic cholestasis.* BMJ Case Rep, 2020. **13**(6).

37. Liu, L.-Y., X.-H. Wang, Y. Lu, et al., *Association of variants of ABCB11 with transient neonatal cholestasis.* Pediatrics International, 2013. **55**(2): p. 138-144.

38. Wang, N.L., L.T. Li, B.B. Wu, et al., *The Features of GGT in Patients with ATP8B1 or ABCB11 Deficiency Improve the Diagnostic Efficiency.* PLoS One, 2016. **11**(4): p. e0153114.

39. Dröge, C., M. Bonus, U. Baumann, et al., *Sequencing of FIC1, BSEP and MDR3 in a large cohort of patients with cholestasis revealed a high number of different genetic variants.* Journal of Hepatology, 2017. **67**(6): p. 1253-1264.

40. Gan, L., S. Pan, J. Cui, et al., *Functional analysis of the correlation between ABCB11 gene mutation and primary intrahepatic stone.* Mol Med Rep, 2019. **19**(1): p. 195-204.

41. Pan, S., X. Li, P. Jiang, et al., *Variations of ABCB4 and ABCB11 genes are associated with primary intrahepatic stones.* Mol Med Rep, 2015. **11**(1): p. 434-446.

42. Kang, H.J., S.A. Hong, S.H. Oh, et al., *Progressive Familial Intrahepatic Cholestasis in Korea: A Clinicopathological Study of Five Patients.* J Pathol Transl Med, 2019. **53**(4): p. 253-260.

43. Hu, G., P. He, Z. Liu, et al., *Diagnosis of ABCB11 gene mutations in children with intrahepatic cholestasis using high resolution melting analysis and direct sequencing.* Mol Med Rep, 2014. **10**(3): p. 1264-1274.

44. Chen, X.-Q., L.-L. Wang, Q.-W. Shan, et al., *Multidrug resistance protein 3 R652G may reduce susceptibility to idiopathic infant cholestasis.* World journal of gastroenterology, 2009. **15**(46): p. 5855-5858.

45. Kim, S.-R., Y. Saito, M. Itoda, et al., *Genetic Variations of the ABC Transporter Gene <i>ABCB11</i> Encoding the Human Bile Salt Export Pump (BSEP) in a Japanese Population.* Drug Metabolism and Pharmacokinetics, 2009. **24**(3): p. 277-281.

46. Goto, K., K. Sugiyama, T. Sugiura, et al., *Bile Salt Export Pump Gene Mutations in Two Japanese Patients With Progressive Familial Intrahepatic Cholestasis.* Journal of Pediatric Gastroenterology and Nutrition, 2003. **36**(5).

47. Sangkhathat, S., W. Laochareonsuk, W. Maneechay, et al., *Variants Associated with Infantile Cholestatic Syndromes Detected in Extrahepatic Biliary Atresia by Whole Exome Studies: A 20-Case Series from Thailand.* Journal of pediatric genetics, 2018. **7**(2): p. 67-73.

48. Davit-Spraul, A., M. Fabre, S. Branchereau, et al., *ATP8B1 and ABCB11 analysis in 62 children with normal gamma-glutamyl transferase progressive familial intrahepatic cholestasis (PFIC): Phenotypic differences between PFIC1 and PFIC2 and natural history.* Hepatology, 2010. **51**(5): p. 1645-1655.

49. van der Woerd, W.L., D.Y. van Haaften-Visser, S.F.J. van de Graaf, et al., *Mutational Analysis of ATP8B1 in Patients with Chronic Pancreatitis.* PLOS ONE, 2013. **8**(11): p. e80553.

50. Goldschmidt, M.L., R. Mourya, J. Connor, et al., *Increased frequency of double and triple heterozygous gene variants in children with intrahepatic cholestasis.* Hepatology Research, 2016. **46**(4): p. 306-311.

51. Togawa, T., T. Sugiura, K. Ito, et al., *Molecular Genetic Dissection and Neonatal/Infantile Intrahepatic Cholestasis Using Targeted Next-Generation Sequencing.* J Pediatr, 2016. **171**: p. 171-7.e1-4.

52. Wang, N.-L., Y. Lu, J.-Y. Gong, et al., *Molecular findings in children with inherited intrahepatic cholestasis.* Pediatric Research, 2020. **87**(1): p. 112-117.

53. Mizutani, A., Y. Sabu, S. Naoi, et al., *Assessment of Adenosine Triphosphatase Phospholipid Transporting 8B1 (ATP8B1) Function in Patients With Cholestasis With ATP8B1 Deficiency by Using Peripheral Blood Monocyte-Derived Macrophages.* Hepatology Communications, 2021. **5**(1): p. 52-62.

54. Klomp, L.W.J., J.C. Vargas, S.W.C. van Mil, et al., *Characterization of mutations in ATP8B1 associated with hereditary cholestasis.* Hepatology, 2004. **40**(1): p. 27-38.

55. Stapelbroek, J.M., K.J. van Erpecum, L.W.J. Klomp, et al., *Nasobiliary drainage induces long-lasting remission in benign recurrent intrahepatic cholestasis.* Hepatology, 2006. **43**(1): p. 51-53.

56. Kulecka, M., A. Habior, A. Paziewska, et al., *Clinical Applicability of Whole-Exome Sequencing Exemplified by a Study in Young Adults with the Advanced Cryptogenic Cholestatic Liver Diseases.* Gastroenterol Res Pract, 2017. **2017**: p. 4761962.

57. Painter, J.N., M. Savander, A. Ropponen, et al., *Sequence variation in the ATP8B1 gene and intrahepatic cholestasis of pregnancy.* European Journal of Human Genetics, 2005. **13**(4): p. 435-439.

58. Zimmer, V., R.M. Bohle, S.N. Weber, et al., *Benign recurrent intrahepatic cholestasis (BRIC)-like episode associated with ATP8B1 variation underlying protracted cholestatic course of acute hepatitis E virus infection.* Dig Liver Dis, 2018. **50**(2): p. 206-207.

59. Ołdakowska-Jedynak, U., I. Jankowska, M. Hartleb, et al., *Treatment of pruritus with Prometheus dialysis and absorption system in a patient with benign recurrent intrahepatic cholestasis.* Hepatol Res, 2014. **44**(10): p. E304-e308.

60. Stolz, A., V. Navarro, P.H. Hayashi, et al., *Severe and protracted cholestasis in 44 young men taking bodybuilding supplements: assessment of genetic, clinical and chemical risk factors.* Alimentary Pharmacology & Therapeutics, 2019. **49**(9): p. 1195-1204.

61. Sambrotta, M., S. Strautnieks, E. Papouli, et al., *Mutations in TJP2 cause progressive cholestatic liver disease.* Nat Genet, 2014. **46**(4): p. 326-8.

62. Liu, X., H. Lai, S. Xin, et al., *Whole-exome sequencing identifies novel mutations in ABC transporter genes associated with intrahepatic cholestasis of pregnancy disease: a case-control study.* BMC pregnancy and childbirth, 2021. **21**(1): p. 110-110.

Current Genes & Health Research Team (in alphabetical order by surname):

Shaheen Akhtar^1^, Mohammad Anwar^2^, Elena Arciero^1^, Samina Ashraf^3^, Saeed Bidi^4^, Gerome Breen^5^, James Broster^4^, Raymond Chung^5^, David Collier^4^, Charles J Curtis^5^, Shabana Chaudhary^4^, Megan Clinch^4^, Grainne Colligan^2^, Panos Deloukas^4^, Ceri Durham^2^, Faiza Durrani^4^, Fabiola Eto^4^, Sarah Finer^4^, Joseph Gafton^4^, Ana Angel Garcia^4^, Chris Griffiths^4^, Joanne Harvey^4^, Teng Heng^1^, Sam Hodgson^4^, Qin Qin Huang^1^, Matt Hurles^1^, Karen A Hunt^4^, Shapna Hussain^4^, Kamrul Islam^4^, Vivek Iyer^1^, Ben Jacobs^4^, Ahsan Khan^4^, Cath Lavery^4^, Sang Hyuck Lee^5^, Robin Lerner^4^, Daniel MacArthur^6^, Daniel Malawsky^1^, Hilary Martin^1^, Dan Mason^7^, Rohini Mathur^4^, Mohammed Bodrul Mazid^4^, John McDermott^8^, Caroline Morton^4^, Bill Newman^8^, Elizabeth Owor^4^, Asma Qureshi^4^, Samiha Rahman^4^, Shwetha Ramachandrappa^5^, Mehru Reza^4^, Jessry Russell^4^, Nishat Safa^4^, Miriam Samuel^4^, Michael Simpson^5^, John Solly^4^, Marie Spreckley^4^, Daniel Stow^4^, Michael Taylor^4^, Richard C Trembath^5^, Karen Tricker^4^, Nasir Uddin^4^, David A van Heel^4^, Klaudia Walter^1^, Caroline Winckley^9^, Suzanne Wood^4^, John Wright^10^, ^11^Julia Zöllner.

^1^Wellcome Sanger Institute, London, UK. ^2^Social Action for Health, London, UK. ^3^Bradford Teaching Hospitals, Bradford, UK. ^4^Queen Mary University of London, London, UK. ^5^King's College London, London, UK. ^6^Garvan Institute of Medical Research, Darlinghurst, Australia. ^7^Born in Bradford, Bradford, UK. ^8^University of Manchester, Manchester, UK. ^9^NIHR Clinical Research Clinical trials, Manchester, UK. ^10^Bradford Institute for Health Research, Bradford, UK. ^11^University College London, London, UK.
